# DEOP: a database on osmoprotectants and associated pathways

**DOI:** 10.1093/database/bau100

**Published:** 2014-10-09

**Authors:** Salim Bougouffa, Aleksandar Radovanovic, Magbubah Essack, Vladimir B. Bajic

**Affiliations:** King Abdullah University of Science and Technology (KAUST); Computational Bioscience Research Centre (CBRC); Computer, Electrical and Mathematical Sciences and Engineering Division (CEMSE); Thuwal, Jeddah, 23955-6900, Saudi Arabia

## Abstract

Microorganisms are known to counteract salt stress through salt influx or by the accumulation of osmoprotectants (also called compatible solutes). Understanding the pathways that synthesize and/or breakdown these osmoprotectants is of interest to studies of crops halotolerance and to biotechnology applications that use microbes as cell factories for production of biomass or commercial chemicals. To facilitate the exploration of osmoprotectants, we have developed the first online resource, ‘Dragon Explorer of Osmoprotection associated Pathways’ (DEOP) that gathers and presents curated information about osmoprotectants, complemented by information about reactions and pathways that use or affect them. A combined total of 141 compounds were confirmed osmoprotectants, which were matched to 1883 reactions and 834 pathways. DEOP can also be used to map genes or microbial genomes to potential osmoprotection-associated pathways, and thus link genes and genomes to other associated osmoprotection information. Moreover, DEOP provides a text-mining utility to search deeper into the scientific literature for supporting evidence or for new associations of osmoprotectants to pathways, reactions, enzymes, genes or organisms. Two case studies are provided to demonstrate the usefulness of DEOP. The system can be accessed at.

**Database URL:** http://www.cbrc.kaust.edu.sa/deop/

## Introduction

Microorganisms need to maintain a stable and a constant cell volume and turgor pressure across their membrane to sustain their metabolic activities. Fluctuations in external salt concentrations would either force the water out of the cell affecting their metabolic activities (1) or cause salt influx into the cytoplasm. Proteins are susceptible to ionic charges, so high concentrations of salt would pull them apart causing their denaturation, consequently arresting the metabolic activities of the microorganism (1).

There are two known strategies used by microorganisms to cope with salt stress. Halophiles, which are microorganisms that thrive in extreme concentrations of salt, use the ‘salt-in-cytoplasm’ mechanism to counteract high salt concentration of the environment. The metabolic machinery is not hindered by the elevated salt concentrations. In fact, these high salt concentrations are required to protect their adapted biomolecules against denaturation. The salt-influx mechanism has so far been reported in a few microorganisms and is not widespread (1, 2).

The majority of microorganisms cope with salt stress by accumulating uncharged, highly soluble organic compounds. These compounds do not interfere with the microbe’s metabolic machinery and hence can be accumulated in large quantities to maintain the equilibrium balance with the outer environment without allowing in large quantities of salt ions. Their solubility and non-interference characteristics are the reason they are named compatible solutes. They are also called osmoprotectants because they protect against salt and osmotic stress. Osmoprotectants include a variety of compound classes, such as sugars (sucrose and trehalose), amino acids (glutamine, proline and alanine), polyols (glycerol, arabitol and inositol) and heterosides (glucosylglycerol and mannosucrose) (1, 2). See Supplementary Table S1 for a list of osmoprotectants.

Osmoprotectants could be accumulated by microorganisms either through direct acquisition from the environment if available or through *de novo* biosynthesis (3). They include a variety of compound classes: sugars and derivatives, amino acids and derivatives and polyols and derivatives (4). Glycine betaine or trimethylglycine is the preferred osmoprotectant in the majority of bacteria (3, 4). Other major osmoprotectants include carnitine and proline. Carnitine is a trimethyl amino acid that is commonly acquired exogenously and accumulated by bacteria under salt stress (5). Proline is an amino acid that can accumulate to high concentrations in bacteria and plants as a response to stress (6, 7). While gram-positive bacteria accumulate proline through enhanced biosynthesis, gram-negative bacteria increase its concentration through uptake from the medium (4).

Among the common sugar osmoprotectants, sucrose and trehalose are accumulated by microorganisms as a response to salt stress. Some of the unusual osmoprotectant sugars include gentiobiose, melibiose, maltose, turanose, raffinose, stachyose, verbascose, altrose, palatinose and cellobiose, which are frequently reported in plants (8, 9). These can also be catabolized to enhance the accumulation of other osmoprotectants. Some of the sugar alcohols (polyols) include glycerol, inositol, mannitol, sorbitol, arabitol and maltitol (10, 11). For detailed reviews of the different classes of osmoprotectants and their mode of action, we refer the reader to published reviews (3, 4, 11–16).

Halotolerant microorganisms have potential applications in biotechnology. Osmoprotectants can act as stabilizers of biomolecules (enzymes, DNA and membranes), whole cells, salt antagonists or salt protective agents. Ectoine and hydroxyectoine are two compatible solutes that were successfully synthesized through biotechnological procedures in a process called ‘bacteria milking’ (17). Cultures of *Halomonas elongata* are subjected to rounds of high and low salt concentrations that cause the biosynthesis of these osmoprotectants under high salt concentration followed by their release into the environment under the low salt concentration. Also, osmoprotection pathways have potential applications in transferring halotolerance to commercially important crops, such as potatoes, wheat, barley and tobacco (17, 18).

Online resources are increasingly available that compile genomic and proteomic information belonging to extremophiles such as HaloWeb (19), HaloBase (20) and Actinobase (21). Despite the availability of pathways databases such as KEGG (22), MetaCyc (23), BioSystems (24) and others, none of them include osmoprotection as a category nor do they dedicate a standardized label for the pathway or compound to highlight its osmoprotective function, other than the occasional reference in the description of some of the well-studied osmoprotectants. To address this gap, we have developed an exploration platform, ‘Dragon Explorer of Osmoprotection associated Pathways’ (DEOP) that focuses on the information related to osmoprotection. This is an online resource that contains curated information on compounds that were reported as osmoprotectants in scientific literature. DEOP provides details of pathways and reactions involving or affecting these compounds with their associated genes and enzymes. However, the shear volume of the rapidly increasing scientific literature prohibits manual curation and discovery of new associations. To that end, this online resource provides a sophisticated text-mining utility that link osmoprotection keywords to the preprocessed NCBI PubMed abstracts, providing support for the current DEOP system. More importantly, it can help discover novel associations. The system is accessible at http://www.cbrc.kaust.edu.sa/deop/. DEOP is free for academic and non-profit use.

## Materials and methods

### Searching for compatible solutes

A comprehensive and accurate search of the scientific literature for osmoprotectants and related pathways is cumbersome and labour-intensive. We simplify the search and information gathering using text mining in the preprocessing stages followed by manual curation. We reduced the PubMed search space by first applying the following keyword combination: osmo* OR organic solute* OR osmol* OR compatible solute* OR stress OR halo* OR salinity OR salt OR rhizosphere OR rhizob* OR brine OR regulatory pathway* OR metabolic pathway*. The search returned 903 563 articles as of the 25 March 2014.

We compiled a non-redundant dictionary file of all chemical compounds and their synonyms from the MetaCyc database. A second dictionary file was compiled that contained the following keywords (one per line): *Compatible solute*(*s*), *osmoprotectant*(*s*), *osmol[iy]te*(*s*), *osmoregulation*, *salt stress* and *stress response*. Co-occurrences in the PubMed database were recorded for terms in the chemical compound dictionary in relation to terms in the second dictionary. For each association in the literature, we calculate the number of articles in which it occurred and store the articles’ Pubmed IDs (PMIDs). We then ranked these associations in a descending order of the number of articles.

The association report narrows down the search scope to those compounds that have been mentioned in the context of osmoprotection or salt stress response. Starting from the associations with the highest article count, we either pull and read the abstracts as per the association PMIDs or re-query PubMed using the following: compound_X AND (osmoly* OR osmoli* OR osmoprot* OR osmoregu* OR compatible solute* OR “salt stress” OR “stress response”).

#### 

##### Classifying a compound as an osmoprotectant

We initially skimmed through abstracts searching for clear reference to the compounds of interest in an osmoprotection context. For example, it was recently reported that *Escherichia coli* protects against osmotic stress by accumulating ubiquinone-8 (Q8) (25). For our purposes, the title of the article (ubiquinone accumulation improves osmotic-stress tolerance in *E. coli*) strongly suggests that Q8 is a true osmoprotectant at least in *E. coli,* and the abstract content confirmed the assumption. We minimize errors in the curation by finding more supporting literature, including full-text articles. Based on the evidence, we classified a compound as an osmoprotectant or a candidate osmoprotectant. We also labelled precursor compounds and compounds involved in osmoregulation according to the literature evidence. A compound is excluded if we fail to assign it any of the labels above.

After we have compiled a curated list of osmoprotectants, we search databases such as BioSystems (24), MetaCyc (23), KEGG (22) for metabolic pathways that produce or consume the compounds. For each instance, we gather available reactions, their directions, the position of the compounds in these reactions, the species in which they were found and the genes and the enzymes involved. Taking a compound-centric approach, we find all reactions that directly impact on the osmoprotectant of interest. We then find the metabolic pathways that involve these reactions. For each pathway, we look for the remaining reactions that complete it. We label osmoprotectants that we could not link to a reaction or a pathway as orphans. Pathways are classified depending on whether the osmoprotectant is produced, is an intermediate or is consumed.

One of the objectives of this work is to provide a mapping facility whereby a gene can be mapped onto an osmoprotection pathway or a genome can be investigated for potential osmoprotection pathways. This requires a comprehensive list of known genes and enzymes involved in the reactions and pathways collected. We used the accession numbers provided by pathway databases such as MetaCyc to download the genomic or protein sequences from sequence databases such as, NCBI Entrez (26), RefSeq (26), Gene (26) and UniProt (27). We convert accession numbers from other databases such as SGD (28), TAIR (29), ExpressArray (30), EcoGene (31) and GenoList (32) to the NCBI Gene ID using the UniProt mapping utility (http://www.uniprot.org/) or by searching the external links on the relevant pages from these databases. We manually checked the consistency of the accession numbers. For downloads from the NCBI databases, we used the Entrez programming utilities (E-utilities) to query and download the sequences of interest. For obsolete sequences, we downloaded the replacement sequences, but often the whole-genome sequence is used as a replacement. In many cases, the gene accession number refers to the full genome too. We dealt with such inconsistencies by first attempting to map to UniProt accession numbers. If a hit is found, we convert the UniProt ID to the NCBI gene ID. If we fail to find a UniProt ID, we search NCBI gene using the gene name or synonyms in combination with the organism name.

The compiled list of genes is used as a BLAST-able backend database. Hits against the database are assigned to the respective pathways. Pathway completeness is estimated by comparing the detected genes against the total number of genes in that pathway. Pathway completeness is a difficult estimate, and therefore, should be used with caution especially when whole genomes are mapped. Genes required for a pathway may differ from one organism to another even for those that may be considered closely related.

## Text-mining methodology

The DEOP literature knowledgebase was compiled using the PubMed biomedical literature database as source. As of April 2014, PubMed lists >23 millions citations from a large scope of fields. We focus our knowledgebase by discarding irrelevant literature. To further narrow the search space, PubMed was queried using a stricter keyword combination: osmo* [title/abstract] OR compatible solute* OR stress [title/abstract] OR haloadapt* OR halophili* OR halotoler* OR salinity OR salt OR rhizosphere OR rhizob* OR brine OR regulatory pathway* OR metabolic pathway*. The query returned 728 162 articles that were downloaded in the XML format for further processing.

**Figure 1.**
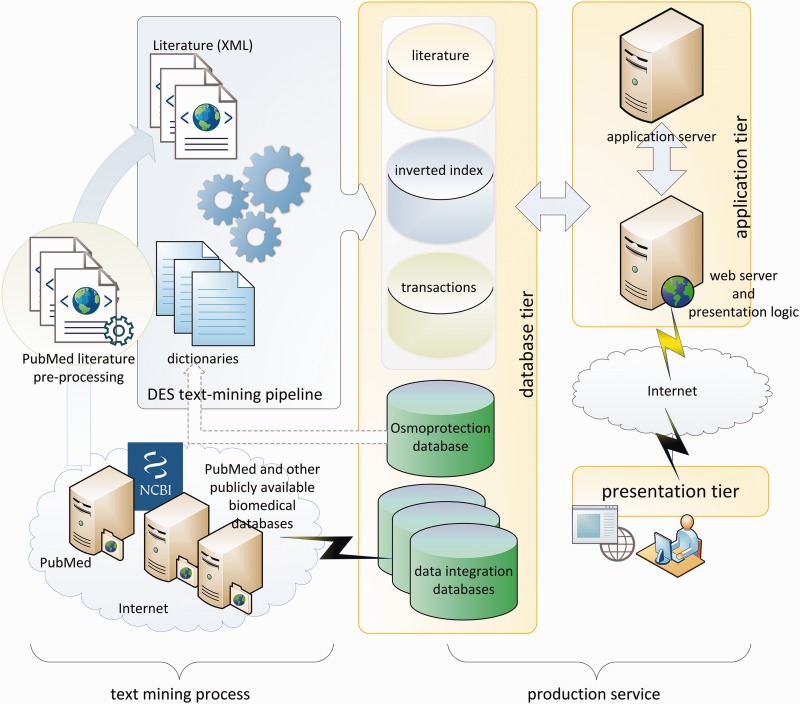
DEOP conceptual diagram.

The text-mining process, as demonstrated in the left panel of Figure 1, is performed by a King Abdullah University of Science and Technology (KAUST)-customized implementation of the dragon exploration system (DES), similar to that in (33). The original DES is a proprietary text-mining and data-mining tool from OrionCell (http://www.orioncell.org). For the purpose of this research, the DES pipeline was redesigned to operate on a high-performance computing cluster. The input for the pipeline is the XML literature corpus generated above and six dictionaries (Table 1) comprising a list of biomedical terms and phrases that we refer to as concepts. These terms or concepts are extracted from the osmoprotectants discovery stage described earlier and include pathway names, reaction Enzyme Comission (EC) numbers, compound names, enzymes, gene names and organisms’ names. The addition of these osmoprotection-specific dictionaries culls the base PubMed corpus and delivers targeted content in a stress-response wide contour whereby related information can be explored and new hypotheses can be made.

**Table 1.** DEOP statistics

Type	Count
Osmoprotectants	109
Orphan osmoprotectants^a^	34
Candidate osmoprotectants^b^	3
Osmoprotection related^c^	23
Other compounds^d^	2738
Pathways	834
Reactions affecting one or more osmoprotectants	1883
Other reactions^e^	2166
Genes	3529
Enzymes	4899
Organisms	1160

^a^An orphan osmoprotectant is one that we could not link to a metabolic pathway.

^b^A candidate osmoprotectant is one that was reported to accumulate in a microorganism under stress but was not labelled as an osmoprotectant.

^c^Compounds that play a role in the osmoprotection response but are not accumulated as osmolytes.

^d^Other compounds in a reaction in which a curated osmoprotectant is involved.

^e^Reactions that are part of a pathway in which one or more osmoprotectants are involved, but these reactions do not directly affect any of the osmoprotectants.

DES text-mining pipeline produced a database of indexes and transactions that are used by production services as shown on the right side of Figure 1. The production services are based on three-tier client/server architecture. The database tier contains the core osmoprotection database, inverted indexes of literature concepts, annotated literature and data integration databases. The application tier consists of application and web servers that execute client requests in the background and prepare the on-screen and downloadable presentations and data. The presentation tier is based on Ajax (34) and jQuery (35) to handle asynchronous requests between clients and servers and to provide the user with feature-rich, smooth and efficient experience.

At the application level, the DEOP system consists of five sets of modules:

1. The osmoprotection database modules

The osmoprotection database modules provide structured access to the osmoprotection PostGres (36) database to execute user-initiated queries, which can be related to pathways, genes/enzymes, compounds, organisms or reactions. The results of these queries are handled by the presentation layer through Ajax calls coupled with jQuery Data Tables (37).

2. The mapping module

The mapping modules are based on the NCBI BLAST program family: blastp (protein vs protein), blastx (translated nucleotide vs protein), blastn (nucleotide vs nucleotide) and tblastn (protein vs translated nucleotide). The default e-value for the BLAST queries is set to 10^−^^4^, but the user can set an e-value of their choice. The mapped pathways are listed for each query sequence along with the hit genes and the number of hits for the concerned pathway. A total summary of the detected pathways is also given, which provides a list of osmoprotectant affected by the pathway, the pathway classification and pathway completeness (%).

3. The literature explorer modules

The literature explorer modules provide concept association reports in textual, spreadsheet or in a graphical format. The reports are assembled from the automatic extraction of the most relevant literature and from document clustering based on content similarity. Contents are evaluated and documents are clustered uzsing self-organizing maps (38, 39). Clustering is performed based on the concepts identified in the documents. For this purpose, we used the program Vsom (40), which is part of the X-window-based microscopy image processing package (41) (see Supplementary Material).

For the researcher, the most valuable module is the “*hypothesis/association explorer*” that is based on Swanson’s ABC model (42). It automatically generates new hypotheses based on the literature content and allows the user to explore and fine-tune the hypotheses via a graphical user interface.

4. The data-mining module

The highly interactive data-mining module allows the user to perform frequent-pattern (FP) mining and association mining. The FP miner discovers concepts that frequently appear in the literature. The methodology is based on the FP-tree structure and the FP-growth method (43). The association miner discovers top associations between concepts by using the TopKRules algorithm (44). Both modules are implemented in the application layer using the ‘Sequential Pattern Mining Framework’ (45).

5. Data enrichment and integration modules

Data enrichment/integration modules include a local MRS-based database system (46), which hosts 31 databases with >77 000 000 records (Table 2) and a database of gene synonyms extracted from the NCBI gene database (Table 3). Biological entities are linked to the MRS search engine to find more information from other databases. By using the overlay design, the user can find more information from the 31 external databases in one place, without the hassle of opening multiple browser windows or tabs.

**Table 2.** Data integration database

Name	Records	Description	Source
Chemical Entities of Biological Interest (ChEBI)	38 580	The database of chemical entities of biological interest	www.ebi.ac.uk/chebi
ChEBI ontology	29 974		
Enzyme	5 418	Enzymes nomencalture	ca.expasy.org/enzyme
Gene	8 927 911	NCBI gene database	www.ncbi.nlm.nih.gov/gene
Functional association data/networks (GeneMania)	21 084	Gene associations database	genemania.org
GO	34 940	Gene ontology database	www.geneontology.org
GOA	11 300 749	Gene ontology annotation	www.ebi.ac.uk/GOA
HUGO Gene nomenclature	35 795	Human genes nomenclature	www.genenames.org
Human major histocompatibility complex	6 939	Human major histocompatibility complex (HLA) sequences	www.ebi.ac.uk/imgt/hla
Immunoglobulins and T-cell receptors nucleotide sequences	156 529	The international imMunoGeneTics information system	www.imgt.org/GeneInfoServlets/htdocs
Interpro	21 749	Protein sequence analysis and classification	www.ebi.ac.uk/interpro
KEGG module	196 659	Collection functional units used for annotation and biological interpretation of sequenced genomes.	www.genome.jp/kegg/module.html
KEGG pathway	262 432	Pathway maps on the molecular interaction and reaction networks for biological interpretation of higher-level systemic functions.	www.genome.jp/kegg/pathway.html
KEGG ligand compound	34 182	Database of chemical substances and reactions that are relevant to life.	www.genome.jp/kegg/ligand.htm
KEGG ligand enzyme	6 118		
KEGG Ligand Glycan	10 985		
KEGG Ligand Reaction	9 400		
Oxford Human Mouse grid	17 834	Laboratory mouse genetic, genomic and biological data resources.	www.informatics.jax.org
Pfam-A	12 273	Collection of protein families, each represented by multiple sequence alignments and hidden Markov models	pfam.sanger.ac.uk
Pfam-B	233 174		
Pfam seed	12 273		
PRINTS	2 050	Protein fingerprints, groups of conserved motifs used to characterize a protein families	bioinf.man.ac.uk/dbbrowser/PRINTS
Prosite	2 247	Documentation entries describing protein domains, families, functional sites and associated patterns and profiles to identify them.	www.expasy.org/prosite/
Prosite documentation	1 621		
REBASE	5 020	The restriction enzyme database.	rebase.neb.com/rebase/rebase.html
RefSeq	18 236 994	Set of reference sequences including genomic, transcript, and protein.	www.ncbi.nlm.nih.gov/refseq/
UniProt/Swiss-Prot	531 473	Protein database, manually annotated and reviewed.	www.uniprot.org/
Taxonomy	817 120	Classification and nomenclature for all of the organisms in the public sequence databases.	www.ncbi.nlm.nih.gov/taxonomy
UniProt/TrEMBL	16 504 022	Protein database, automatically annotated and not reviewed.	www.uniprot.org
Unigene	2 652 777	NCBI database of the transcriptome.	www.ncbi.nlm.nih.gov/unigene
Uniprot KB	17 035 495	Protein knowledgebase (Swiss-Prot + TrEMBL).	www.uniprot.org
Total records	77 163 817

**Table 3.** Synonyms extracted from NCBI Gene database

Synonym type	Number of synonyms
Alias	756 689
Alternate_name	405 135
Gene_Symbol	1 366 580
Locus_tag	9 824 390
Official_Full_Name	109 969
Official_Symbol	296 280
Total synonyms	12 759 043

## Backend information

The database is hosted on a PostgreSQL server v.9.3. The web front of the database was coded in PHP. Mapping of genes/genomes is handled using a combination of PHP and Perl scripts.

## Results and discussion

To date, we are not aware of a resource that is dedicated to providing detailed and curated information about osmoprotectant compounds, complemented by relevant pathways, reactions, enzymes, genes and organisms. DEOP addresses this gap. DEOP is a hybrid online resource of human-based curation and computational-based compilation. The foundation of the resource is the manually curated list of osmoprotectants. We built on the list by adding reactions that produce or consume these compounds, and then added the pathways and involved genes and proteins. A rich text-mining and cross-referencing layer was then added to enrich the knowledge base. For the current statistics of the database, refer to Table 1.

## Access to the resource

Access to the database is facilitated by means of combined browsing and searching. The database has five entry points depending on the object of interest. The database can be browsed for compounds, pathways, reactions, enzymes, their genes and organisms. In each browser page, data are represented in tabular format with Ajax case-insensitive search boxes on top of each column, which can be used to filter the content in their respective columns. Multiple search boxes can be populated simultaneously for combination searches. We also provide an all-columns search box that filters content in all of the columns, which can also be used in combination with the other column-specific filters.

To facilitate browsing, the tables are paginated with only 10 rows showing on the first page. Columns can also be sorted alphabetically by clicking on the column title. A left click on the text in the cells activates context menus that are content-dependent. For example, a left-click on a pathway name on the pathways page provides three menu options: ‘MRS cross-reference’, ‘Literature References’ and ‘Pathway Details’.

The MRS cross-reference context menu searches our large MRS-based databank (Table 2) using the selected keyword. Instead of supplying limiting external links to identical biological entities in other databases, a user is able to retrieve the same information and more without having to navigate away from the current page.

The literature reference menu links the biological entity of interest, e.g. a compound or a gene, to the preprocessed PubMed abstracts. The report highlights, using colour, the current biological term and other relevant terms (if found) in the abstracts. This provides a comprehensive, current and content-specific literature support that is expected to save time and effort and even unearth new information.

Further details are provided for each type of biological data through the context menu. The details page for a pathway lists its osmoprotection classification, affected compounds, participating reactions and host organisms. For a compound, the resource displays the formula, an interactive Jmol-based 3D structure (47), ChEBI synonyms (48), charge, molecular mass, pathways it is involved in and producing and consuming reactions. For genes, the resource provides the gene name, synonyms and the name of the protein product, whereas a reaction detail page lists the EC number if available, the entailing pathways and organisms and the catalysing enzymes. Finally, an organism’s detail page lists its specific information.

Despite the existence of curated and well-established metabolic pathway databases, none provide standardized osmoprotection labelling. With the mapping tool that we developed, the utility of our resource is extended beyond mere listing of biological terms. It takes genomic or protein sequences and maps them to the pathways using NCBI BLAST programs. The stringency of the mapping can be adjusted by tuning the minimum bit score and the minimum alignment length and/or the percentage identity. Pathway completeness is calculated based on the number of gene hits to a particular pathway divided by the total number of genes in that pathway. This completeness measure can assist the user to decide whether a pathway really exists in their data.

## The text-mining modules

Manual curation is labour-intensive and time-consuming especially considering the amount of publications and the rate of publications. A significant part of DEOP is the ‘Literature Explorer’ module based on osmoprotection-related literature available from the PubMed. The module consists of a number of sub-modules: literature statistics, literature relevance, document clustering and association reports. In addition, various download services are linked to the modules that allow the user to get data in spreadsheet forms and analyse them offline. The text-mining modules are supported by two data-mining modules aimed for association and FP discovery.

The literature statistics report gives a broad overview of available literature. It summarizes the results of the text-mining process by giving information about the number of relevant abstracts and number of recognized biomedical concepts available in the system. In addition, charts showing two types of distribution are displayed: (i) distribution of dictionaries and (ii) frequency of concepts regardless of dictionary. This information can be used as a quick reference, for example, to see the number of pathways mentioned or described in the literature. Related to this overview is the literature relevance report that actualizes the statistical overview into annotated text available for reading. Abstracts are sorted by frequency of terms found in them, and can be viewed as annotated text and in original form. In the annotated text, concepts belonging to different dictionaries are highlighted using different colours to help the user to focus on certain biomedical concepts with the ability to switch on and off one or more dictionaries. This useful feature is available throughout the system. More sophisticated literature relevance overview is given in the document clustering report. Documents are evaluated using a self-organizing map. The algorithm uses the frequency of concepts identified in the documents. The weight of a feature equals its frequency in the cluster divided by the total number of documents in the cluster. The clustering report offers the top five clusters that have >400 documents.

For a researcher, one of the most useful modules is perhaps the association report. Concepts found in the literature are grouped according to the dictionaries and then cross-referenced. This feature allows the user to browse or search through the list of concepts, discover associated concepts, generate and visually explore association networks and hypotheses and download customized reports in the form of spreadsheets. Each concept is enriched by a plethora of additional information hosted on our local MRS database that is a click away.

The underlying DES hypothesis discovery module is based on Swanson’s work (42, 49) and on the open-discovery approach (50, 51), but it goes a step further. The main problem with automatic hypotheses creation is the enormous number of generated hypotheses; most represent concepts associated by simple co-occurrence but with no biological meaning. Our system is interactive in which the user begins with a chosen concept and progresses towards hypotheses by manually selecting dictionaries and their underlying concepts. In each step, the system offers a list of hypotheses derived from the osmoprotection literature and tested by querying the whole PubMed.

We implemented two data-mining algorithms to text mine the database. The first is the FP miner, which discovers patterns of concepts that frequently appear together in the literature. For example, results show that the concept glutathione appears with the concept oxygen in 6375 documents (1.3% of all documents). The number of documents in which one or more terms co-occur is called support. The user can adjust the support using a percentage slider to choose the minimum support number while searching for frequent patterns.

The second data-mining module is the association miner, which discovers the probability of the co-occurrence of various concepts in the literature. For example, we noted that the combination of concepts alanine, rat and aspartate occurs together in 682 or in 0.14% of all documents. In other words, the supporting literature for this rule is 0.14%. In the remaining literature, those concepts may occur in different binary combinations, singles or not at all. We also observed that if a document contains the combination of concepts alanine and rat, there is a 65% chance of finding aspartate. This association can be formulated as follows: the occurrence of alanine and rat implies the occurrence of aspartate with 65% confidence. The association miner is an interactive tool that provides the user sliders to choose the number of top associations as well as the minimum confidence.

## Case study 1

To show how DEOP can be used to extract useful knowledge about osmoprotectants, we performed a case study to identify osmoprotection-related pathways in three microbes that were isolated from the Red Sea Shaban Deep and compared them with six other microbial genomes that were isolated from normal sea waters in other locations. We downloaded the predicted proteins of the following eight bacteria and an archaeon from INDIGO (52): *Betaproteobacteria KB13* (BioProject: PRJNA54261, the OM43 clade), *Methylophilales* bacterium HTCC2181 (BioProject: PRJNA54577, OM43 clade), ‘*Candidatus Pelagibacter ubique* HTCC1062’ (BioProject: PRJNA58401, SAR11), ‘*Candidatus Pelagibacter sp. HTCC7211*’ (BioProject: PRJNA54705, SAR11), ‘*Candidatus Pelagibacter* sp. IMCC9063’ (BioProject: PRJNA66305, SAR11), the alphabacterium IMCC14465 strain (BioProject: PRJNA174247) *Haloplasma contractile* SSD-17B (BioProject: PRJNA67925), *Salinisphaera shabanensis* E1L3A (BioProject: PRJNA67923) and *Halorhabdus tiamatea* str. SARL4B (BioProject: PRJNA67927, *Archaea*). SSD-17B, E1L3A and SARL4B were isolated and sequenced from the Shaban Deep (53–55). The two bacteria were isolated from the brine-seawater interface, whereas the archaeon was isolated from the brine-sediment interface.

**Figure 2.**
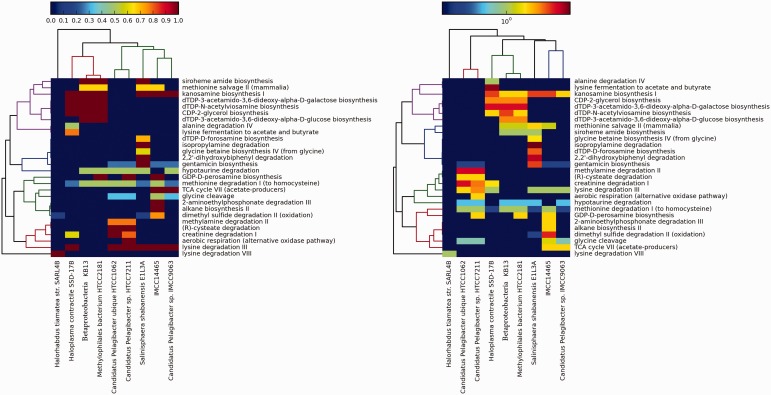
Values in the left panel are pathway completeness estimates. The values in the right panel are the total hits for each pathway that was normalized against its completeness. A minimum completeness of 50% was applied to both panels, where at least one sample had to satisfy for each pathway. The left panel demonstrates the presence or absence of selected pathways in the mangrove samples vs the control data sets, whereas the right panel shows the enrichment of these pathways.

First, we mapped the predicted proteins using BLAST programs (we initially used an e-value of 10^−^^5^, then we filtered the results using a stricter bit score of 50 and a minimum percentage identity of 60%) against our gene data set. Then, hits were assigned to the various pathways. We compared the genomes against each other for pathways presence/absence and completeness and enrichment. We only included pathways that were at least 50% complete. Two heatmaps were generated: (i) for the first heatmap, we used the completeness values that range from 0 to 1 as an approximate binary estimate of the presence or absence of pathways (Figure 2, left panel). (ii) For the right panel, we used the log of the normalized total number of hits to a pathway as a measure of enrichment. We performed normalization by multiplying the number of hits to a pathway by its completeness value.

In both panels of Figure 2, the bacteria form a sub-cluster that is separated from the archaeon *H. tiamatea* str. SARL4B. Within the bacterial sub-cluster, the OM43 bacteria KB13 and HTCC2181 and *H. contractile* SSD-17B contain several complete pathways that synthesize L-glutamate as an intermediate or final product: dTDP-3-acetamido-3, 6-dideoxy-alpha-D-galactose biosynthesis, dTDP-3-acetamido-3, 6-dideoxy-alpha-D-glucose biosynthesis, siroheme amide biosynthesis and dTDP-N-acetylviosamine biosynthesis. The CDP-2-glycerol biosynthesis pathway, which synthesizes glycerol (a known osmoprotectant), was also solely found in these three bacteria. Unique to *H. contractile* SSD-17B are the pathways alanine degradation IV and lysine fermentation to acetate and butyrate that synthesize the osmoprotectants L-alanine and L-lysine as side products.

*S. shabanensis* E1L3A uniquely contained the following pathways: glycine betaine biosynthesis from glycine IV (produces the universal osmoprotectant glycine betaine), 2,2’-dihydroxybiphenyl degradation (produces the osmoregulator salicylate as a by-product of aromatic compound degradation), isopropylamine degradation (degrades the osmoprotectant L-glutamate but synthesizes L-alanine that acts as an osmoprotectant in some microorganisms) and dTDP-D-forosamine biosynthesis (synthesizes L-glutamate as an intermediate product). Interestingly, the archaeon *H. tiamatea* str. SARL4B lacked all of the bacterial pathways suggesting that this archaeon copes with the harsh conditions at the sediment-brine interface using different mechanisms; perhaps the salt-in-cytoplasm strategy. In summary, the purpose of this brief comparison is to demonstrate the utility of DEOP in study adaptation strategy at the genomic level.

## Case study 2

**Figure 3.**
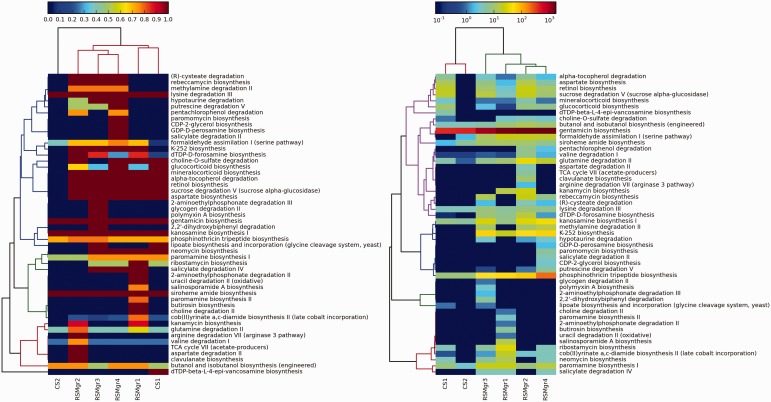
Values in the left panel are pathway completeness estimates. The values in the right panel are the total hits for each pathway that was normalized against its completeness. A minimum completeness of 75% was applied to both panels, where at least one sample had to satisfy for each pathway. The left panel demonstrates the presence or absence of selected pathways in the mangrove samples vs the control data sets, whereas the right panel shows the enrichment of these pathways.

In the second case study, we demonstrate the utility of DEOP in investigating potential osmoprotection-related pathways in six 454-pyrosequenced metagenomic data sets collected from KAUST shores on the Red Sea (data will be published in a separate study). Four of the metagenomic data sets are for the mangrove *Avicennia marina* rhizosphere (RSMgr1-4), whereas the other two are for neighbouring control sediment (CS1-2). Artificially duplicated reads were removed using CD-HIT-454 with default settings (56). We used the NGS QC toolkit (57) to discard reads that contained five or more ambiguous bases as well as those that contained eight or more homopolymer stretches. A minimum length cut-off of 150 bp was also applied. The successful reads were BLAST-ed against the DEOP gene database (Bit score 50, minimum percentage identity 60%) and mapped to DEOP pathways. We limited the downstream analysis to pathways that were 75% complete or higher. Two heatmaps were also generated using the method explained above (Figure 3).

The mangrove samples clustered together in both panels in Figure 3, although the control sample CS01 did not separate from the mangrove sub-cluster in the left panel. Some of the pathways that were unique to the mangrove samples include (R)-cysteate degradation (L-glutamate, intermediate product), rebeccamycin biosynthesis (β-D-glucose degraded), methylamine degradation II (L-glutamate and N-methyl-L-glutamate, degraded), hypotaurine degradation (hypotaurine consumed and L-alanine degraded), putrescine degradation V (4-aminobutanoate intermediate, L-alanine product, putrescine reversibly degraded), K-252 biosynthesis (dimethylglycine and glycine betaine, degraded), DTDP-D-forosamine biosynthesis (L-glutamate intermediate), choline-O-sulfate degradation (Choline, product) and aspartate biosynthesis (L-aspartate and L-glutamate, products).

K-252, rebeccamycin, candicidin and oleandomycin are all antibiotics produced from actinomycetes, whereas dTDP-D-desosamine is an essential component of macrolide antibiotics revealing the availability of the osmolytes used in these pathways, that provide osmoprotection, and using these osmolytes to produce antibiotics provides an additional advantage of eliminating competitors that do not have resistance to them.

Putrescine is a mangrove exudate that has been reported to impact the rhizophere microbiome as increased uptake of putrescine causes decreased growth rates and consequently decreased ability for competitive colonisation (58); but putrescine is also known to play an important role in protecting against osmotic stress (59). So, the presence of putrescine degradation pathways is probably a mechanism to produce other osmolytes, while at the same time keep higher growth rates.

## Conclusion

DEOP is an online resource that gathers literature verified osmoprotectants and lists all available pathways and reactions that affect them. Through the genes associated with the pathways, we are able to predict the presence of these pathways in organisms with available genomic sequences. Although, the osmoprotectants listed on the resource were reported in the literature as such, the detection of a pathway in a genome by computational methods is not conclusive evidence for its activation or its role in osmoprotection, as biodiversity is incredibly big and many organisms can, under abiotic stress, use some pathways and not others despite the presence of all. The causes of this selectivity are unknown, although the environment is the prime suspect. Having said this, the resource will facilitate the assignment of genomic or protein sequences to the pathways that impact on osmoprotectant compounds. The application of such resource is evident in studies interested in abiotic stress responses and plant stress tolerance, as well as industries interested in microbes as cell factories.

## Supplementary data

Supplementary data are available at *Database* Online.

Supplementary Data
