# Supplementary material for: DEOP: a database on osmoprotectants and associated pathways
Source: Database (Oxford). 2014 Oct 9;2014:bau100. doi: 10.1093/database/bau100 (PMC4201361; doi:10.1093/database/bau100)
Supplement: Supplementary Data [file supp_2014_bau100_index.html]

DEOP: a database on osmoprotectants and associated pathways — Supplementary Data 

# DEOP: a database on osmoprotectants and associated pathways

## Supplementary Data

files

**Files in this Data Supplement:**

- Supplementary Data - doc file
- Supplementary Data - doc file
