# Supplementary material for: DEOP: a database on osmoprotectants and associated pathways
Source: Database (Oxford). 2014 Oct 9;2014:bau100. doi: 10.1093/database/bau100 (PMC4201361; doi:10.1093/database/bau100)
Supplement: Supplementary Data [file supp_bau100_Supplemental_Methods.doc]

**The self-organising maps**

The algorithm described in (1) maps a set of n-dimensional vectors onto a two-dimensional array of nodes. Vectors are created from 72,250 concepts, found in 903,563 abstracts, used as features in the clustering algorithm. The weight of a feature equals to its frequency in the cluster divided by the total number of documents in the cluster. The clustering algorithm implementation in we used is an utility (Vsom, http://xmipp.cnb.csic.es/NewXmipp/Web_Site/public_html/Xmipp/chapter8/vsom.html), from the X-Window-based Microscopy Image Processing Package (2).

1. Marabini, R., Carazo, J.M. (1994) Pattern recognition and classification of images of biological macromolecules using artificial neural networks. *Biophysical journal*, **66**, 1804-1814.

2. de la Rosa-Trevín, J.M., Otón, J., Marabini, R.*, et al.* (2013) Xmipp 3.0: An improved software suite for image processing in electron microscopy. *Journal of Structural Biology*, **184**, 321-328.
