# Supplementary material for: DEOP: a database on osmoprotectants and associated pathways
Source: Database (Oxford). 2014 Oct 9;2014:bau100. doi: 10.1093/database/bau100 (PMC4201361; doi:10.1093/database/bau100)
Supplement: Supplementary Data [file supp_bau100_Supplemental_Tables_v2.doc]

Table S1: Literature curated list of osmoprotectants

| **Name** | **Formula** | **Role** | **Organisms** |
| --- | --- | --- | --- |
| dimethylsulfonioacetate | C4H8O2S1 | • Osmoprotectant |  |
| dimethyl sulfoxide | C2H6O1S1 | • Osmoprotectant | • Acinetobacter sp. strain 20B • Alcaligenes sp. strain E1 • Arthrobacter methylotrophus • Arthrobacter sulfonivorans • Escherichia coli • Halobacterium sp. NRC-1 • Hyphomicrobium denitrificans • Hyphomicrobium sp. EG • Hyphomicrobium sp. S • Hyphomicrobium sulfonivorans • Pseudomonas putida • Rhodococcus sp. strain SY1 • Rhodovulum sulfidophilum • Thiocapsa roseopersicina |
| ectoine | C6H10N2O2 | • Osmoprotectant | • Chromohalobacter salexigens |
| gamma-butyrobetaine | C7H15N1O2 | • Osmoprotectant | • Agrobacterium • Escherichia coli • Homo sapiens • Mus musculus • Neurospora crassa • Pseudomonas aeruginosa • Pseudomonas putida • Pseudomonas sp. AK-1 • Rattus norvegicus • Xanthomonas translucens |
| hypotaurine | C2H7N1O2S1 | • Osmoprotectant | • Bos taurus • Homo sapiens • Mammalia • Mus musculus • Paracoccus denitrificans • Rattus norvegicus |
| L-dehydro-ascorbate | C6H6O6 | • Osmoprotectant | • Amanita muscaria • Apis mellifera • Arabidopsis thaliana • Bos taurus • Brassica rapa • Caenorhabditis elegans • Capsicum annuum • Cavia porcellus • Chlamydomonas reinhardtii • Cucumis melo • Cucumis sativus • Cucurbita pepo medullosa  • Dianthus caryophyllus • Drosophila melanogaster • Dugesia japonica • Escherichia coli • Euglena • Glycine max • Homo sapiens • Klebsiella pneumoniae • Lactuca sativa romaine • Malus domestica • Mus musculus • Nicotiana plumbaginifolia • Paul's Scarlet Climber Rose • Pelargonium crispum • Periplaneta americana • Pisum sativum • Rattus norvegicus • Solanum lycopersicum • Solanum tuberosum • Spinacia oleracea • Vigna radiata • Xanthophyllomyces dendrorhous |
| L-pipecolate | C6H11N1O2 | • Osmoprotectant | • Pseudomonas fluorescens • Pseudomonas putida |
| methylnicotinate | C7H7N1O2 | • Osmoprotectant | • Glycine max • Pisum sativum |
| N-acetyl-L-2,4-diaminobutanoate | C6H12N2O3 | • Osmoprotectant | • Chromohalobacter salexigens |
| L-Ndelta-acetylornithine | C7H14N2O3 | • Osmoprotectant | • Arabidopsis thaliana |
| glutathione disulfide | C20H30N6O12S2 | • Osmoprotectant | • Allochromatium vinosum • Arabidopsis thaliana • Astragalus • Bos taurus • Brassica juncea • Brassica rapa • Cavia porcellus • Chlorella sp. (Roon 68) • Cucumis sativus • Cucurbita pepo medullosa  • Drosophila melanogaster • Escherichia coli • Euglena • Glycine max • Homo sapiens • Lemna minor • Mus musculus • Neptunia • Oryctolagus cuniculus • Phanerochaete chrysosporium • Pisum sativum • Populus tremula x Populus alba • Pseudomonas cepacia • Pseudomonas sp GU5 • Rattus norvegicus • Saccharomyces cerevisiae • Solanum tuberosum • Sphingobium chlorophenolicum • Sphingobium japonicum • Sphingomonas sp. SYK6 • Spinacia oleracea |
| quinine | C20H25N2O2 | • Osmolyte-Transport Regulation • Non-Osmolyte | • Cinchona • Cinchona calisaya • Cinchona macrocalyx • Cinchona mutisii • Cinchona officinalis • Cinchona pitayensis • Cinchona pubescens |
| dimethylsulfoniopropanoate | C5H10O2S1 | • Osmoprotectant | • Alcaligenes faecalis M3A • Aspergillus sydowii • Burkholderia ambifaria • Candidatus Pelagibacter ubique • Clostridium propionicum • Crypthecodinium cohnii • Desulfobacterium sp. PM4 • Desulfobacterium vacuolatum • Desulfococcus niacini • Desulfovibrio acrylicus • Dinoroseobacter shibae • DSMP-degrading bacterium LFR • Emiliania huxleyi • Fulvimarina pelagi • Fusarium lateritium • gamma proteobacterium HTCC2207 • Labrenzia aggregata • Loktanella vestfoldensis • Marinomonas sp. MED121 • Marinomonas sp. MWYL1 • Maritimibacter alkaliphilus • Melosira nummuloides • Oceanicola batsensis • Oceanimonas doudoroffii • Polysiphonia paniculata • Rhodobacter sphaeroides • Roseovarius nubinhibens • Ruegeria atlantica • ruegeria frisia • Ruegeria pomeroyi • Sagittula stellata • Sinorhizobium fredii • Spartina alterniflora • Sulfitobacter sp. EE-36 • Sulfitobacter sp. NAS-14.1 • Tetraselmis • Ulva curvata • Ulva intestinalis • Ulva lactuca • Vertebrata lanosa • Wedelia biflora |
| trimethylamine N-oxide | C3H9N1O1 | • Osmoprotectant | • Aminobacter aminovorans • Escherichia coli • Methylocella silvestris • Methyloversatilis universalis • Methylovorus mays |
| urea | C1H4N2O1 | • Osmoprotectant • Osmoregulation | • Achromobacter denitrificans • Agrobacterium tumefaciens • Alcaligenes sp. • Arabidopsis thaliana • Arthrobacter globiformis • Arthrobacter sp. KUJ8602 • Arthrobacter sp. TE1826 • Avena sativa • Bacillus caldovelox • Bacillus fastidiosus • Bacillus licheniformis • Bacillus subtilis • Bos taurus • Brassica juncea • Brevibacterium helvolum • Brucella melitensis • Canavalia ensiformis • Canavalia lineata • Capsicum annuum • Chlamydomonas reinhardtii • Chlorella sp. (Roon 68) • Cyberlindnera jadinii • Datura stramonium • Dianthus caryophyllus • Enterobacter aerogenes • Escherichia coli • Glycine max • Gordonia rubripertincta • Helicobacter pylori • Homo sapiens • Leishmania donovani • Malus domestica • Mus musculus • Mycobacterium phlei • Mycobacterium smegmatis • Nicotiana tabacum • Oleomonas sagaranensis • Oryza sativa • Phaseolus vulgaris • Pimelobacter simplex • Pinus sylvestris • Pisum sativum • Pseudomonas aeruginosa • Pseudomonas putida • Pseudomonas sp. Ps7 • Pseudomonas stutzeri • Rattus norvegicus • Rhizobiaceae • Rhodococcus erythropolis • Saccharomyces cerevisiae • Sinorhizobium meliloti Rm2011 • Solanum lycopersicum • Streptococcus salivarius • Streptomyces clavuligerus • Streptomyces griseus • Streptomyces violaceochromogenes • Sulfolobus solfataricus • Synechocystis sp. PCC 6803 • Theobroma cacao • Thermococcus kodakarensis • Thermus aquaticus • Thermus thermophilus • Tissierella creatinini • Tissierella creatinophila |
| 2-(beta-D-glucosyl)-sn-glycerol | C9H18O8 | • Osmoprotectant |  |
| 4-aminobutanoate | C4H9N1O2 | • Osmoprotectant | • Arabidopsis thaliana • Arthrobacter globiformis • Arthrobacter nicotinovorans • Arthrobacter sp. • Arthrobacter sp. KUJ8602 • Bacillus subtilis • Brevibacterium helvolum • Clostridium aminobutyricum • Cupriavidus necator • Enterobacter aerogenes • Escherichia coli • Homo sapiens • Kocuria rosea • Leishmania donovani • Mammalia • Mus musculus • Nocardioides sp. JS614 • Pimelobacter simplex • Pisum sativum • Pseudomonas aeruginosa • Pseudomonas fluorescens • Pseudomonas putida • Ralstonia eutropha • Rattus norvegicus • Rhodococcus opacus • Saccharomyces cerevisiae • Solanum lycopersicum • Streptomyces griseus • Streptomyces violaceochromogenes |
| sym-homospermidine | C8H24N3 | • Osmoprotectant | • Nicotiana tabacum • Senecio vernalis • Senecio vulgaris |
| alpha-D-galactosyl-(1,1')-sn-glycerol 3-phosphate | C9H17O11P1 | • Osmoprotectant |  |
| 2-(alpha-D-galactosyl)-sn-glycerol 3-phosphate | C9H17O11P1 | • Osmoregulation • Candidate |  |
| hexane-1,3,4,6-tetracarboxylate | C10H10O8 | • Osmoprotectant | • Archaeoglobus fulgidus • Methanobacteria • Methanobrevibacter • Methanocaldococcus jannaschii • Methanococci • Methanoculleus • Methanogenium • Methanosarcina • Methanothermobacter thermautotrophicus • Methanothermus |
| stachydrine | C7H13N1O2 | • Osmoprotectant | • Sinorhizobium meliloti Rm2011 |
| 5-oxoproline | C5H6N1O3 | • Osmoprotectant | • Bacillus circulans • Homo sapiens • Musca domestica • Rattus • Rattus norvegicus • Schizosaccharomyces pombe |
| acetylcholine | C7H16N1O2 | • Osmoprotectant |  |
| guanidinium | C1H6N3 | • Osmoprotectant • Osmoregulation | • Penicillium digitatum • Pseudomonas syringae • Saccharomyces cerevisiae |
| betaine aldehyde | C5H12N1O1 | • Osmoprotectant | • Arabidopsis thaliana • Arthrobacter globiformis • Arthrobacter pascens • Arthrobacter sp. • Bacillus subtilis • Chromohalobacter salexigens • Cylindrocarpon didymum • Escherichia coli • Fusarium anguioides • Fusarium Caucasicum  • Fusarium fujikuroi • Fusarium oxysporum • Homo sapiens • Hordeum vulgare • Pseudomonas aeruginosa • Pseudomonas putida • Sinorhizobium meliloti Rm2011 • Spinacia oleracea |
| choline | C5H14N1O1 | • Osmoprotectant | • Agrobacterium fabrum • Agrobacterium tumefaciens • Arabidopsis thaliana • Arthrobacter globiformis • Arthrobacter pascens • Arthrobacter sp. • Bacillus subtilis • Borrelia burgdorferi • Brassica napus • Brucella abortus • Catharanthus roseus • Chromohalobacter salexigens • Cylindrocarpon didymum • Desulfovibrio alaskensis • Escherichia coli • Fluoribacter bozemanae • Fusarium anguioides • Fusarium Caucasicum  • Fusarium fujikuroi • Fusarium oxysporum • Glycine max • Homo sapiens • Hordeum vulgare • Legionella pneumophila • Mus musculus • Pisum sativum • Pseudomonas aeruginosa • Pseudomonas putida • Raphanus sativus • Ricinus communis • Saccharomyces cerevisiae • Sinorhizobium meliloti • Sinorhizobium meliloti Rm2011 • Spinacia oleracea • Treponema denticola • Triticum aestivum |
| citrate | C6H5O7 | • Precursor | • Acetobacter aceti • Acidithiobacillus thiooxidans • Aquifex aeolicus • Aquifex pyrophilus • Arabidopsis thaliana • Aspergillus itaconicus • Aspergillus terreus • Azotobacter vinelandii • Bacillus anthracis • Bacillus cereus • Bacillus clausii • Bacillus thuringiensis • Candidatus Arcobacter sulfidicus • Chlorobaculum tepidum • Chlorobaculum thiosulfatiphilum • Chlorobium limicola • Clostridium pasteurianum • Cyanobium gracile • Desulfobacter hydrogenophilus • Dickeya dadantii • Enterobacter aerogenes • Enterococcus faecalis • Erwinia chrysanthemi • Escherichia coli • Euglena gracilis • Geminocystis herdmanii • Glycine max • Haloarcula marismortui • Haloferax volcanii • Helicobacter pylori • Homo sapiens • Hydrogenobacter thermophilus • Klebsiella pneumoniae • Marinobacter aquaeolei • Marinobacter hydrocarbonoclasticus • Methylobacterium extorquens • Methylobacterium populi • Mycobacterium bovis • Mycobacterium smegmatis • Mycobacterium tuberculosis • Natrialba magadii • Phaeobacter gallaeciensis • Photobacterium angustum • Photobacterium sp. SKA34 • Pseudomonas syringae • Pyrobaculum islandicum • Pyrobaculum neutrophilum • Rhodopseudomonas palustris • Rubrivivax gelatinosus • Saccharomyces cerevisiae • Salmonella enterica • Shigella boydii • Shigella flexneri • Sinorhizobium meliloti Rm2011 • Streptococcus mutans • Sulfurimonas denitrificans • Synechococcus elongatus • Synechococcus sp. PCC 7002 • Synechocystis sp. PCC 6803 • Thermoproteus tenax • Thiobacillus thioparus • Vigna radiata • Yersinia frederiksenii • Yersinia intermedia • Yersinia kristensenii |
| creatine-phosphate | C4H8N3O5P1 | • Osmoprotectant | • Gallus gallus • Homo sapiens • Oryctolagus cuniculus |
| glycine betaine | C5H11N1O2 | • Osmoprotectant | • Actinopolyspora halophila • Agrobacterium • Aliivibrio fischeri • Aphanothece halophytica • Arabidopsis thaliana • Arthrobacter • Arthrobacter globiformis • Arthrobacter pascens • Arthrobacter sp. • Bacillus subtilis • Camellia irrawadiensis • Camellia ptilophylla • Camellia sinensis • Catharanthus roseus • Chromohalobacter salexigens • Coffea abeokutae • Coffea arabica • Coffea eugenioides • Coffea liberica • Corynebacterium sp. • Cylindrocarpon didymum • Emiliania huxleyi • Erythroxylum coca • Escherichia coli • Fusarium anguioides • Fusarium Caucasicum  • Fusarium fujikuroi • Fusarium oxysporum • Halorhodospira halochloris • Homo sapiens • Hordeum vulgare • Lupinus luteus • Melosira nummuloides • Methanohalophilus portucalensis • Mycobacterium tuberculosis • Nocardiopsis sp. K-290 • Nonomuraea longicatena • Peganum harmala • Porphyromonas gingivalis • Pseudomonas aeruginosa • Pseudomonas putida • Pseudomonas sp. AK-1 • Rattus norvegicus • Saccharomyces cerevisiae • Salmonella enterica • Sinorhizobium meliloti Rm2011 • Sorghum bicolor • Spinacia oleracea • Tetraselmis • Ulva intestinalis • Ulva lactuca • Vibrio cholerae • Vibrio harveyi • Xanthomonas translucens |
| dimethylglycine | C4H9N1O2 | • Osmoprotectant | • Actinopolyspora halophila • Aliivibrio fischeri • Aphanothece halophytica • Arabidopsis thaliana • Arthrobacter • Bacillus subtilis • Camellia irrawadiensis • Camellia ptilophylla • Camellia sinensis • Catharanthus roseus • Coffea abeokutae • Coffea arabica • Coffea eugenioides • Coffea liberica • Corynebacterium sp. • Emiliania huxleyi • Erythroxylum coca • Escherichia coli • Halorhodospira halochloris • Homo sapiens • Lupinus luteus • Melosira nummuloides • Methanohalophilus portucalensis • Mycobacterium tuberculosis • Nocardiopsis sp. K-290 • Nonomuraea longicatena • Peganum harmala • Porphyromonas gingivalis • Pseudomonas aeruginosa • Pseudomonas putida • Rattus norvegicus • Saccharomyces cerevisiae • Salmonella enterica • Sinorhizobium meliloti Rm2011 • Sorghum bicolor • Tetraselmis • Ulva intestinalis • Ulva lactuca • Vibrio cholerae • Vibrio harveyi |
| arachidonate | C20H31O2 | • Non-Osmolyte • Cell-Wall Modifications Under Salt Stress | • Acinetobacter sp. ADP1 • Anabaena variabilis • Arabidopsis thaliana • Botryococcus braunii • Candida cloacae • Candida tropicalis • Carthamus tinctorius • Cucumis sativus • Cyanothece sp. ATCC 51142 • Cyanothece sp. PCC 7425 • Escherichia coli • Gloeobacter violaceus • Glycine max • Homo sapiens • Jeotgalicoccus sp. ATCC 8456 • Lycopersicon hirsutum • Marchantia polymorpha • Micrococcus luteus • Mortierella alpina • Mus musculus • Nicotiana tabacum • Nostoc punctiforme • Oryza sativa • Petunia x hybrida • Physcomitrella patens • Pisum sativum • Prochlorococcus marinus • Saccharomyces cerevisiae • Solanum lycopersicum • Solanum tuberosum • Synechococcus elongatus • Synechocystis sp. PCC 6803 • Zymomonas mobilis |
| palmitate | C16H31O2 | • Osmoprotectant | • Arabidopsis thaliana • Brassica napus • Candida cloacae • Candida tropicalis • Carthamus tinctorius • Cucumis sativus • Escherichia coli • Glycine max • Homo sapiens • Lycopersicon hirsutum • Micrococcus luteus • Mus musculus • Nicotiana tabacum • Oryza sativa • Pisum sativum • Rattus norvegicus • Saccharomyces cerevisiae • Solanum lycopersicum • Solanum tuberosum • Spinacia oleracea |
| L-carnitine | C7H15N1O3 | • Osmoprotectant | • Agrobacterium • Bos taurus • Escherichia coli • Homo sapiens • Mus musculus • Neurospora crassa • Ovis aries • Pseudomonas aeruginosa • Pseudomonas putida • Pseudomonas sp. AK-1 • Rattus norvegicus • Sus scrofa • Xanthomonas translucens |
| trans-aconitate | C6H3O6 | • Osmoprotectant | • Escherichia coli • Lycopersicon hirsutum • Micrococcus luteus • Saccharomyces cerevisiae • Solanum lycopersicum |
| L-aspartate | C4H6N1O4 | • Osmoprotectant | • Acidithiobacillus thiooxidans • Acinetobacter sp. ADP1 • Amaranthus cruentus • Amaranthus hypochondriacus • Arabidopsis thaliana • Archaeoglobus fulgidus • Arenicola marina • Arthrobacter globiformis • Ascaris lumbricoides • Ascaris suum • Azotobacter vinelandii • Bacillus cereus • Bacillus circulans • Bacillus licheniformis • Bacillus megaterium • Bacillus mycoides • Bacillus pumilus • Bacillus subtilis • Bacteroides thetaiotaomicron • Bordetella pertussis • Bos taurus • Brevibacillus laterosporus • Brevibacterium • Campylobacter jejuni • Canavalia ensiformis • Canavalia lineata • Chlamydia trachomatis • Chlamydomonas • Chromohalobacter salexigens • Citrus sinensis • Corbicula japonica • Corynebacterium glutamicum • Crassostrea gigas • Cryptococcus albidus • Cupriavidus metallidurans • Cyanobium gracile • Cylindrocarpon obtusisporum • Cytophaga hutchinsonii • Deinococcus radiodurans • Emiliania huxleyi • Enterobacter aerogenes • Erwinia chrysanthemi • Escherichia coli • Fasciola hepatica • Geminocystis herdmanii • Geobacillus stearothermophilus • Glycine max • Gossypium hirsutum • Haemophilus influenzae • Halalkalicoccus • Haloarcula • Haloferax • Haloferax mediterranei • Halomicrobium • Haloterrigena • Helicobacter pylori • Homo sapiens • Hordeum vulgare • Ignicoccus • Klebsiella oxytoca • Klebsiella pneumoniae • Linum usitatissimum • Lupinus albus • Lupinus angustifolius • Lupinus luteus • Lysinibacillus sphaericus • Malus domestica • Megathyrsus maximus • Melosira nummuloides • Metallosphaera sedula • Methanobacteria • Methanobrevibacter • Methanocaldococcus jannaschii • Methanococci • Methanoculleus • Methanogenium • Methanosarcina • Methanothermobacter thermautotrophicus • Methanothermus • Moritella sp. • Musca domestica • Mycobacterium tuberculosis • Mytilus californianus • Mytilus edulis • Mytilus galloprovincialis • Mytilus trossulus • Myxococcus xanthus • Natrialba • Natronomonas • Neisseriaceae • Neurospora crassa • Nicotiana rustica • Nicotiana tabacum • Oryza sativa • Paenibacillus macerans • Paenibacillus polymyxa • Panicum miliaceum • Pectobacterium carotovorum • Penicillium digitatum • Pisum sativum • Plantago major • Populus tremula x Populus tremuloides • Prevotella ruminicola • Proteus vulgaris • Pseudoalteromonas haloplanktis • Pseudomonas • Pseudomonas aeruginosa • Pseudomonas putida • Pyrobaculum • Ralstonia solanacearum • Rattus • Rattus norvegicus • Rhodospirillum rubrum • Ricinus communis • Saccharomyces cerevisiae • Salmonella enterica • Schizosaccharomyces pombe • Sipunculus nudus • Solanum lycopersicum • Solanum tuberosum • Sorghum bicolor • Sphaerobacter • Spinacia oleracea • Sporosarcina globispora • Sporosarcina pasteurii • Streptomyces griseus • Streptomyces rimosus • Sulfolobus acidocaldarius • Sulfolobus solfataricus • Sus scrofa • Synechocystis • Synechocystis sp. PCC 6803 • Tannerella forsythia • Tetraselmis • Thermobaculum • Thermomicrobium • Thermoproteus • Thermosynechococcus elongatus • Thermotoga neapolitana • Thermus aquaticus • Thermus thermophilus • Thiobacillus thioparus • Ulva intestinalis • Ulva lactuca • Urochloa panicoides • Vibrio alginolyticus • Vibrio cholerae • Vibrionaceae • Vibrio parahaemolyticus • Vibrio vulnificus • Vicia faba • Virgibacillus pantothenticus • Wedelia biflora • Xanthomonas axonopodis • Xanthomonas campestris • Xylella fastidiosa • Zea mays |
| (S)-malate | C4H4O5 | • Osmoprotectant | • Acetobacter aceti • Acidithiobacillus thiooxidans • Acinetobacter calcoaceticus • Amaranthus cruentus • Amaranthus hypochondriacus • Aquifex aeolicus • Aquifex pyrophilus • Arabidopsis thaliana • Arenicola marina • Ascaris lumbricoides • Ascaris suum • Azotobacter vinelandii • Bacillus subtilis • Brassica napus • Candidatus Arcobacter sulfidicus • Chlorobaculum tepidum • Chlorobaculum thiosulfatiphilum • Chlorobium limicola • Chloroflexus aggregans • Chloroflexus aurantiacus • Clostridium pasteurianum • Corbicula japonica • Crassostrea gigas • Cucumis sativus • Cyanobium gracile • Desulfobacter hydrogenophilus • Desulfobulbus propionicus • Enterococcus faecalis • Escherichia coli • Euglena gracilis • Fasciola hepatica • Geminocystis herdmanii • Glycine max • Haloarcula marismortui • Haloferax volcanii • Helicobacter pylori • Homo sapiens • Hordeum vulgare • Hydrogenobacter thermophilus • Hyphomicrobium methylovorum • Hyphomicrobium zavarzinii • Lycopersicon hirsutum • Megathyrsus maximus • Methanococcus maripaludis • Methanospirillum hungatei • Methanothermobacter thermautotrophicus • Methylobacterium extorquens • Methylobacterium organophilum • Methylobacter whittenburyi • Methylocystis echinoides • Methylocystis minimus • Methylocystis parvus • Methylocystis pyriformis • Methylosinus sporium • Methylosinus trichosporium • Micrococcus luteus • Mycobacterium bovis • Mycobacterium smegmatis • Mycobacterium tuberculosis • Mytilus californianus • Mytilus edulis • Mytilus galloprovincialis • Mytilus trossulus • Natrialba magadii • Nicotiana tabacum • Oryza sativa • Panicum miliaceum • Paracoccus versutus • Pectinatus frisingensis • Pelobacter propionicus • Pisum sativum • Propionibacterium acidipropionici • Propionibacterium acnes • Propionibacterium cyclohexanicum • Propionibacterium freudenreichii • Pyrobaculum islandicum • Pyrobaculum neutrophilum • Raphanus sativus • Rhodobacter capsulatus • Rhodobacter sphaeroides • Rhodospirillum rubrum • Ricinus communis • Roseiflexus castenholzii • Roseiflexus sp. RS-1 • Saccharomyces cerevisiae • Sipunculus nudus • Solanum lycopersicum • Solanum tuberosum • Streptococcus mutans • Streptomyces coelicolor • Sulfolobus acidocaldarius • Sulfolobus solfataricus • Sulfurimonas denitrificans • Sus scrofa • Synechococcus sp. PCC 7002 • Thermoproteus tenax • Thiobacillus thioparus • Trifolium pratense • Urochloa panicoides • Vicia faba • Vigna radiata • Zea mays |
| salicylate | C7H5O3 | • Osmoregulation • Non-Osmolyte | • Arabidopsis lyrata • Arabidopsis thaliana • Brassica napus • Burkholderia cepacia • Catharanthus roseus • Clarkia breweri • Cucumis sativus • Fagopyrum esculentum • Ipomoea nil • Mycobacterium tuberculosis • Nicotiana tabacum • Populus • Pseudomonas aeruginosa • Pseudomonas fluorescens • Pseudomonas nitroreducens • pseudomonas putida • Pseudomonas putida • Pseudomonas reinekei • Pseudomonas sp. ATCC 17483 • Pseudomonas sp. NCIB9816-4 • Pseudomonas sp. PG • Pseudomonas stutzeri • Ralstonia sp. U2 • Rhodococcus sp. NCIMB12038 • Sphingomonas wittichii • Streptomyces sp. WA46 • Trichosporon cutaneum • Trichosporon moniliiforme • Yersinia enterocolitica • Yersinia pestis • Yersinia pseudotuberculosis |
| glycerol | C3H8O3 | • Osmoprotectant | • Aquifex aeolicus • Arabidopsis thaliana • Aspergillus nidulans • Aspergillus niger • Bacillus subtilis • Burkholderia cenocepacia • Citrobacter freundii • Clostridium acetobutylicum • Clostridium butyricum • Clostridium pasteurianum • Deinococcus radiodurans • Enterobacter aerogenes • Escherichia coli • Glycine max • Haemophilus influenzae • Ipomoea batatas • Klebsiella pneumoniae • Lactobacillus brevis • Lactobacillus buchneri • Medicago truncatula • Nicotiana tabacum • Pseudomonas aeruginosa • Saccharomyces cerevisiae • Schizosaccharomyces pombe • Solanum lycopersicum • Solanum tuberosum • Streptococcus pneumoniae • Synechocystis • Thermotoga maritima • Trichoderma reesei • Zea mays |
| D-mannitol | C6H14O6 | • Osmoprotectant | • Apium graveolens • Aquifex aeolicus • Arabidopsis thaliana • Bacillus subtilis • Burkholderia cenocepacia • Cassia coluteoides • Clostridium acetobutylicum • Cryptosporidium parvum • Deinococcus radiodurans • Eimeria • Eimeria tenella • Escherichia coli • Glycine max • Haemophilus influenzae • Ipomoea batatas • Medicago truncatula • Nicotiana tabacum • Pseudomonas aeruginosa • Solanum lycopersicum • Solanum tuberosum • Synechocystis • Thermotoga maritima • Toxoplasma gondii • Zea mays |
| D-sorbitol | C6H14O6 | • Osmoprotectant | • Aquifex aeolicus • Arabidopsis thaliana • Aspergillus nidulans • Aspergillus niger • Bacillus subtilis • Burkholderia cenocepacia • Clostridium acetobutylicum • Deinococcus radiodurans • Escherichia coli • Gluconobacter • Glycine max • Haemophilus influenzae • Ipomoea batatas • Ketogulonicigenium vulgare DSM 4025 • Malus domestica • Medicago truncatula • Nicotiana tabacum • Pseudomonas aeruginosa • Pyrus pyrifolia • Solanum lycopersicum • Solanum tuberosum • Synechocystis • Thermotoga maritima • Trichoderma reesei • Zea mays • Zymomonas mobilis |
| maltitol | C12H24O11 | • Osmoprotectant | • Aquifex aeolicus • Arabidopsis thaliana • Bacillus subtilis • Burkholderia cenocepacia • Clostridium acetobutylicum • Deinococcus radiodurans • Escherichia coli • Glycine max • Haemophilus influenzae • Ipomoea batatas • Medicago truncatula • Nicotiana tabacum • Pseudomonas aeruginosa • Solanum lycopersicum • Solanum tuberosum • Synechocystis • Thermotoga maritima • Zea mays |
| erythritol | C4H10O4 | • Osmoprotectant | • Aquifex aeolicus • Arabidopsis thaliana • Bacillus subtilis • Burkholderia cenocepacia • Clostridium acetobutylicum • Deinococcus radiodurans • Escherichia coli • Glycine max • Haemophilus influenzae • Ipomoea batatas • Medicago truncatula • Nicotiana tabacum • Pseudomonas aeruginosa • Solanum lycopersicum • Solanum tuberosum • Synechocystis • Thermotoga maritima • Zea mays |
| L-arabitol | C5H12O5 | • Osmoprotectant | • Aquifex aeolicus • Arabidopsis thaliana • Aspergillus niger • Aspergillus oryzae • Bacillus subtilis • Burkholderia cenocepacia • Clostridium acetobutylicum • Deinococcus radiodurans • Escherichia coli • Glycine max • Haemophilus influenzae • Ipomoea batatas • Medicago truncatula • Nicotiana tabacum • Penicillium chrysogenum • Pseudomonas aeruginosa • Solanum lycopersicum • Solanum tuberosum • Synechocystis • Thermotoga maritima • Trichoderma reesei • Zea mays |
| xylitol | C5H12O5 | • Osmoprotectant | • Aquifex aeolicus • Arabidopsis thaliana • Aspergillus niger • Aspergillus oryzae • Bacillus subtilis • Bos taurus • Burkholderia cenocepacia • Cavia porcellus • Clostridium acetobutylicum • Deinococcus radiodurans • Escherichia coli • Glycine max • Haemophilus influenzae • Homo sapiens • Ipomoea batatas • Medicago truncatula • Mesocricetus auratus • Morganella morganii • Mus musculus • Nicotiana tabacum • Oryctolagus cuniculus • Penicillium chrysogenum • Providencia stuartii • Pseudomonas aeruginosa • Rattus norvegicus • Serratia marcescens • Solanum lycopersicum • Solanum tuberosum • Sus scrofa • Synechocystis • Thermotoga maritima • Trichoderma reesei • Zea mays |
| 1D-chiro-inositol | C6H12O6 | • Osmoprotectant | • Aquifex aeolicus • Arabidopsis thaliana • Bacillus subtilis • Beta vulgaris • Burkholderia cenocepacia • Clostridium acetobutylicum • Deinococcus radiodurans • Escherichia coli • Fagopyrum esculentum • Glycine max • Haemophilus influenzae • Ipomoea batatas • Lupinus albus • Lupinus luteus • Medicago truncatula • Nicotiana tabacum • Pseudomonas aeruginosa • Simmondsia chinensis • Solanum lycopersicum • Solanum tuberosum • Synechocystis • Thermotoga maritima • Zea mays |
| myo-inositol | C6H12O6 | • Osmoprotectant | • Aquifex aeolicus • Arabidopsis thaliana • Archaeoglobus fulgidus • Avena sativa • Bacillus subtilis • Bacteroides thetaiotaomicron • Burkholderia cenocepacia • Cerastium arvense • Clostridium acetobutylicum • Cucurbita pepo • Deinococcus radiodurans • Dictyostelium discoideum • Escherichia coli • Glycine max • Haemophilus influenzae • Homo sapiens • Hordeum vulgare • Ipomoea batatas • Lens culinaris • Lilium longiflorum • Limnanthes douglasii • Lupinus luteus • Medicago truncatula • Mesembryanthemum crystallinum • Methanocaldococcus jannaschii • Mycobacterium smegmatis • Mycobacterium tuberculosis • Nicotiana tabacum • Novosphingobium aromaticivorans • Oryza sativa • Pantoea agglomerans • Phaseolus vulgaris • Pisum sativum • Pseudomonas aeruginosa • Pseudomonas syringae • Rattus norvegicus • Ricinus communis • Saccharomyces cerevisiae • Secale cereale • Solanum lycopersicum • Solanum tuberosum • Spirodela polyrhiza • Stellaria media • Streptomyces bikiniensis • Streptomyces galbus • Streptomyces griseus • Streptomyces ornatus • Sulfolobus solfataricus • Synechocystis • Thermotoga maritima • Thermotoga neapolitana • Trifolium incarnatum • Triticum aestivum • Triticum spelta • Vicia faba • Vigna angularis • Vigna radiata • Zea mays |
| galactinol | C12H22O11 | • Osmoprotectant | • Arabidopsis thaliana • Beta vulgaris • Cerastium arvense • Cucurbita pepo • Fagopyrum esculentum • Glycine max • Lens culinaris • Lupinus albus • Lupinus luteus • Phaseolus vulgaris • Pisum sativum • Simmondsia chinensis • Solanum lycopersicum • Stellaria media • Vicia faba • Vigna angularis |
| L-quebrachitol | C7H14O6 | • Osmoprotectant | • Aquifex aeolicus • Arabidopsis thaliana • Bacillus subtilis • Burkholderia cenocepacia • Clostridium acetobutylicum • Deinococcus radiodurans • Escherichia coli • Glycine max • Haemophilus influenzae • Ipomoea batatas • Medicago truncatula • Nicotiana tabacum • Pseudomonas aeruginosa • Solanum lycopersicum • Solanum tuberosum • Synechocystis • Thermotoga maritima • Zea mays |
| D-pinitol | C7H14O6 | • Osmoprotectant | • Aquifex aeolicus • Arabidopsis thaliana • Bacillus subtilis • Burkholderia cenocepacia • Clostridium acetobutylicum • Deinococcus radiodurans • Escherichia coli • Glycine max • Haemophilus influenzae • Ipomoea batatas • Lens culinaris • Medicago truncatula • Mesembryanthemum crystallinum • Nicotiana tabacum • Pseudomonas aeruginosa • Solanum lycopersicum • Solanum tuberosum • Synechocystis • Thermotoga maritima • Trifolium incarnatum • Vigna angularis • Zea mays |
| D-ononitol | C7H14O6 | • Osmoprotectant | • Aquifex aeolicus • Arabidopsis thaliana • Bacillus subtilis • Burkholderia cenocepacia • Clostridium acetobutylicum • Deinococcus radiodurans • Escherichia coli • Glycine max • Haemophilus influenzae • Ipomoea batatas • Lens culinaris • Medicago truncatula • Mesembryanthemum crystallinum • Nicotiana tabacum • Pseudomonas aeruginosa • Solanum lycopersicum • Solanum tuberosum • Synechocystis • Thermotoga maritima • Vigna angularis • Zea mays |
| L,L-di-myo-inositol 1,3'-phosphate | C12H22O14P1 | • Osmoprotectant | • Aeropyrum pernix • Aquifex aeolicus • Arabidopsis thaliana • Archaeoglobus fulgidus • Bacillus subtilis • Burkholderia cenocepacia • Clostridium acetobutylicum • Deinococcus radiodurans • Escherichia coli • Glycine max • Haemophilus influenzae • Hyperthermus butylicus • Ipomoea batatas • Medicago truncatula • Nicotiana tabacum • Pseudomonas aeruginosa • Pyrococcus furiosus • Pyrococcus woesei • Rubrobacter xylanophilus • Solanum lycopersicum • Solanum tuberosum • Synechocystis • Thermococcus kodakarensis • Thermotoga maritima • Zea mays |
| alpha-D-galactose | C6H12O6 | • Osmoprotectant | • Arabidopsis thaliana • Cucumis melo • Cucumis sativus • Escherichia coli • Glycine max • Mycoplasma pneumoniae • Oryza sativa • Streptococcus thermophilus • Vibrio sp. JT0107 • Vicia faba • Zobellia galactanivorans |
| alpha-D-mannose | C6H12O6 | • Osmoprotectant | • Apium graveolens • Bacteroides thetaiotaomicron • Cassia coluteoides • Oncorhynchus mykiss |
| beta-D-mannose | C6H12O6 | • Osmoprotectant | • Apium graveolens • Bacteroides thetaiotaomicron • Cassia coluteoides • Oncorhynchus mykiss |
| beta-D-glucose | C6H12O6 | • Osmoprotectant | • Acetivibrio cellulolyticus • Adlercreutzia equolifaciens • Agapanthus africanus • Agaricus bisporus • Agrobacterium tumefaciens • Allium cepa • Aloe arborescens • Apis mellifera • Arabidopsis thaliana • Asaccharobacter celatus • Asanoa ferruginea • Aspergillus aculeatus • Aspergillus japonicus • Aspergillus niger • Aspergillus oryzae • Bacillus subtilis • [Bacteroides] cellulosolvens • Bifidobacterium adolescentis • Bifidobacterium animalis • Bifidobacterium bifidum • Bifidobacterium longum • Bradyrhizobium japonicum • Brassica napus • Caulobacter vibrioides • Celosia cristata • Chlamydomonas reinhardtii • Chrysosporium lucknowense • Cicer arietinum • Cichorium intybus • Clitoria ternatea • Clostridium acetobutylicum • Clostridium cellulolyticum • Clostridium cellulovorans • Clostridium josui • Clostridium papyrosolvens • Clostridium thermocellum • Corynebacterium sp. SHS 0007 • Dalbergia cochinchinensis • Daphne odora • Delphinium grandiflorum • Dianthus caryophyllus • Drosophila melanogaster • Enterococcus faecalis • Enterococcus faecium • Escherichia coli • Euglena gracilis • Fagopyrum esculentum • Fagopyrum tataricum • Flammulina velutipes • Fragaria • Fragaria x ananassa • Geobacillus stearothermophilus • Giardia intestinalis • Gluconobacter oxydans • Glycine max • Grifola frondosa • Haloarcula marismortui • Halococcus saccharolyticus • Haloferax mediterranei • Halorubrum saccharovorum • Helianthus tuberosus • Hevea brasiliensis • Homo sapiens • Hordeum vulgare • human intestinal bacterium SNU-Julong732 • Klebsiella pneumoniae • Kocuria varians • Lactobacillus acidophilus • Lactobacillus brevis • Lactobacillus buchneri • Lactobacillus casei • Lactobacillus delbrueckii • Lactobacillus dextrinicus • Lactobacillus fermentum • Lactobacillus plantarum • Lactobacillus reuteri • Lactobacillus salivarius • Lactobacillus sanfranciscensis • Lactococcus garvieae • Lactococcus lactis • Lampranthus sociorum • Lechevalieria aerocolonigenes • Lentinus sajor-caju • Lepidium sativum • Leuconostoc lactis • Leuconostoc mesenteroides • Linum usitatissimum • Lotus japonicus • Manduca sexta • Manihot esculenta • Medicago sativa • Medicago truncatula • Melilotus albus • Microbacterium sp. • Neisseria meningitidis • Nicotiana tabacum • Oenococcus oeni • Oryza sativa • Oxybasis rubra • Paecilomyces sp. • Paenibacillus popilliae • Panax ginseng • Pantoea agglomerans • Pantoea cypripedii • Pediococcus acidilactici • Pediococcus damnosus • Phytophthora cactorum • Picea abies • Pichia fermentans • Pinus banksiana • Pinus contorta • Pinus strobus • Pisum sativum • Plesiomonas sp. Yoshida 95 • Pleurotus ostreatus • Populus deltoides • Prunus serotina • Pseudomonas aeruginosa • Pseudomonas fluorescens • Pseudomonas putida • Pyrococcus abyssi • Pyrococcus furiosus • Pyrococcus horikoshii • Pyrococcus woesei • Pythium irregulare • Quercus robur • Quercus rubra • Raphanus sativus • Rattus norvegicus • Rauvolfia serpentina • Rhodococcus sp. • Rhus typhina • Rosa • Ruminococcus albus • Saccharomyces cerevisiae • Schizophyllum commune • Scytonema sp. • Secale cereale • Serratia marcescens • Slackia isoflavoniconvertens • Slackia sp. NATTS • Solanum lycopersicum • Solanum tuberosum • Sorghum bicolor • Spinacia oleracea • Staphylococcus aureus • Streptococcus equinus • Streptococcus mutans • Streptococcus thermophilus • Streptomyces antibioticus • Sulfolobus acidocaldarius • Sulfolobus solfataricus • Sulfolobus tokodaii • Sus scrofa • Thermoanaerobacter brockii • Thermococcus litoralis • Thermoplasma acidophilum • Thermoproteus tenax • Thermotoga maritima • Trichoderma reesei • Triglochin maritima • Weissella confusa • Weissella paramesenteroides • Zea mays • Zymomonas mobilis |
| alpha-D-glucose | C6H12O6 | • Osmoprotectant | • Agaricus bisporus • Agrobacterium tumefaciens • Apis mellifera • Arabidopsis thaliana • Asanoa ferruginea • Bacillus subtilis • Bradyrhizobium japonicum • Chlamydomonas reinhardtii • Corynebacterium sp. SHS 0007 • Crambe hispanica • Dalbergia cochinchinensis • Escherichia coli • Euglena gracilis • Flammulina velutipes • Geobacillus stearothermophilus • Gluconobacter oxydans • Grifola frondosa • Haloarcula marismortui • Halococcus saccharolyticus • Haloferax mediterranei • Halorubrum saccharovorum • Homo sapiens • Hordeum vulgare • Klebsiella pneumoniae • Kocuria varians • Lentinus sajor-caju • Microbacterium sp. • Nicotiana tabacum • Oryza sativa • Paecilomyces sp. • Paenibacillus popilliae • Panax ginseng • Pantoea agglomerans • Pantoea cypripedii • Phytophthora cactorum • Pichia fermentans • Plesiomonas sp. Yoshida 95 • Pleurotus ostreatus • Pseudomonas aeruginosa • Pseudomonas fluorescens • Pseudomonas putida • Pyrococcus furiosus • Pyrococcus horikoshii • Pythium irregulare • Raphanus sativus • Saccharomyces cerevisiae • Schizophyllum commune • Scytonema sp. • Serratia marcescens • Solanum tuberosum • Spinacia oleracea • Sulfolobus acidocaldarius • Sulfolobus solfataricus • Sulfolobus tokodaii • Thermoanaerobacter brockii • Thermococcus litoralis • Thermococcus sp. B1001 • Thermoplasma acidophilum • Thermoproteus tenax • Zea mays • Zymomonas mobilis |
| aldehydo-D-altrose | C6H12O6 | • Osmoprotectant |  |
| (S)-acetoin | C4H8O2 | • Osmoprotectant | • Corynebacterium glutamicum • Enterobacter aerogenes • Geobacillus stearothermophilus • Klebsiella pneumoniae • Saccharomyces cerevisiae |
| (R)-acetoin | C4H8O2 | • Osmoprotectant | • Aeromonas hydrophila • Bacillus subtilis • Brevibacillus brevis • Corynebacterium glutamicum • Enterobacter aerogenes • Geobacillus stearothermophilus • Gluconobacter oxydans • Klebsiella pneumoniae • Lactobacillus brevis • Lactobacillus casei • Lactobacillus helveticus • Lactobacillus plantarum • Lactococcus lactis • Leuconostoc lactis • Leuconostoc mesenteroides • Oenococcus oeni • Ogataea angusta • Paenibacillus polymyxa • Pectobacterium carotovorum • Pediococcus pentosaceus • Raoultella terrigena • Saccharomyces cerevisiae • Serratia marcescens |
| alpha-tocopherol | C29H50O2 | • Osmoprotectant | • Arabidopsis thaliana • Homo sapiens • Rattus norvegicus • Synechocystis sp. PCC 6803 |
| ubiquinone-8 | C49H74O4 | • Osmoprotectant | • Acetobacter aceti • Acidithiobacillus ferrooxidans • Acidithiobacillus thiooxidans • Acinetobacter sp. ADP1 • Agrobacterium tumefaciens • Alcaligines sp. • Allochromatium vinosum • Aphanothece halophytica • Arabidopsis thaliana • Aspergillus niger • Avena sativa • Azotobacter vinelandii • Bos taurus • Bradyrhizobium japonicum • Burkholderia sp. WS • Capsicum annuum • Chlorobaculum tepidum • Chlorobium limicola • Corynebacterium sp. SHS 0007 • Cyanobium gracile • Deinococcus radiodurans • Escherichia coli • Euglena gracilis • Geminocystis herdmanii • Gluconobacter oxydans • Glycine max • Haloarcula marismortui • Homo sapiens • Klebsiella pneumoniae • Lactuca sativa romaine • Mus musculus • Mycobacterium bovis • Mycobacterium smegmatis • Mycobacterium tuberculosis • Natrialba magadii • Nicotiana tabacum • Nitrosomonas europaea • Oryza sativa • Pantoea agglomerans • Pantoea cypripedii • Paracoccus denitrificans • Pectobacterium carotovorum • Photobacterium leiognathi • Photorhabdus luminescens • Pseudanabaena limnetica • Pseudomonas aeruginosa • Pseudomonas fluorescens • Pseudomonas putida • Pseudomonas sp. CBB1 • Rattus norvegicus • Rhodobacter capsulatus • Saccharomyces cerevisiae • Salmonella enterica • Serratia marcescens • Shewanella putrefaciens • Sinorhizobium meliloti Rm2011 • Solanum lycopersicum • Solanum tuberosum • Spinacia oleracea • Synechococcus elongatus • Synechococcus sp. PCC 7002 • Synechocystis • Thiobacillus thioparus • Trypanosoma brucei • Typhonium venosum • Vigna radiata • Zea mays • Zymomonas mobilis |
| agmatine | C5H16N4 | • Candidate | • Aeromonas caviae • Arabidopsis thaliana • Avena sativa • Bacillus subtilis • Brassica juncea • Campylobacter jejuni • Capsicum annuum • Datura stramonium • Dianthus caryophyllus • Escherichia coli • Glycine max • Homo sapiens • Hordeum vulgare • Malus domestica • Mycobacterium phlei • Mycobacterium smegmatis • Nicotiana tabacum • Oryza sativa • Pisum sativum • Pseudomonas aeruginosa • Solanum lycopersicum • Theobroma cacao • Thermococcus kodakarensis • Thermus thermophilus |
| spermidine | C7H22N3 | • Osmoprotectant | • Arabidopsis • Arabidopsis thaliana • Archaea • Aves • Bacillus anthracis • Bacillus cereus • Bacillus clausii • Bacillus subtilis • Bacillus thuringiensis • Bos taurus • Campylobacter jejuni • Crithidia fasciculata • Escherichia coli • Eukaryota • Geobacillus stearothermophilus • Homo sapiens • Mammalia • Marinobacter aquaeolei • Marinobacter hydrocarbonoclasticus • Methylobacterium extorquens • Methylobacterium populi • Mus musculus • Nicotiana tabacum • Phaeobacter gallaeciensis • Photobacterium angustum • Photobacterium sp. SKA34 • Physarum polycephalum • Pseudomonas aeruginosa • Rhodopseudomonas palustris • Saccharomyces cerevisiae • Salmonella enterica • Senecio vernalis • Senecio vulgaris • Solanum lycopersicum • Sulfolobus solfataricus • Teleostei • Thermococcus kodakarensis • Thermus thermophilus • Vibrio alginolyticus • Vibrio cholerae • Vibrio parahaemolyticus • Vibrio vulnificus • Zea mays |
| putrescine | C4H14N2 | • Osmoprotectant | • Achromobacter xylosoxidans • Aeromonas caviae • Arabidopsis thaliana • Archaea • Arthrobacter sp. • Atropa belladonna • Avena sativa • Bacillus subtilis • Bordetella bronchiseptica • Bordetella parapertussis • Bordetella pertussis • Bos taurus • Brassica juncea • Campylobacter jejuni • Capsicum annuum • Datura stramonium • Dianthus caryophyllus • Enterobacter aerogenes • Escherichia coli • Eukaryota • Glycine max • Homo sapiens • Hyoscyamus albus • Hyoscyamus niger • Kocuria rosea • Malus domestica • Mammalia • Mus musculus • Mycobacterium phlei • Mycobacterium smegmatis • Nicotiana tabacum • Oryza sativa • Pisum sativum • Pseudomonas aeruginosa • Rattus norvegicus • Saccharomyces cerevisiae • Senecio vernalis • Senecio vulgaris • Shewanella putrefaciens • Shewanella sp. MR-4 • Shewanella sp. MR-7 • Solanum lycopersicum • Solanum tuberosum • Theobroma cacao • Thermococcus kodakarensis • Vibrio alginolyticus • Vibrio cholerae • Vibrio parahaemolyticus • Vibrio vulnificus |
| L-citrulline | C6H13N3O3 | • Osmoprotectant | • Aeromonas caviae • Aphanocapsa • Arabidopsis thaliana • Bacillus subtilis • Bacteroides thetaiotaomicron • Bos taurus • Chlamydomonas • [Clostridium] sticklandii • Corynebacterium glutamicum • Cytophaga hutchinsonii • Escherichia coli • Giardia intestinalis • Glycine max • Halalkalicoccus • Haloarcula • Halobacterium salinarum • Haloferax • Halomicrobium • Haloterrigena • Hexamita inflata • Homo sapiens • Ignicoccus • Lactobacillus hilgardii • Lactobacillus plantarum • Metallosphaera sedula • Methanothermobacter thermautotrophicus • Moritella sp. • Mus musculus • Mycoplasma hominis • Mycoplasma pneumoniae • Myxococcus xanthus • Natrialba • Natronomonas • Neisseriaceae • Neurospora crassa • Oenococcus oeni • Prevotella ruminicola • Pseudomonas • Pseudomonas aeruginosa • Pyrobaculum • Rattus norvegicus • Saccharomyces cerevisiae • Salmonella enterica • Solanum lycopersicum • Sphaerobacter • Spiroplasma citri • Streptococcus ratti • Sulfolobus acidocaldarius • Tannerella forsythia • Tetrahymena pyriformis • Thermobaculum • Thermomicrobium • Thermoproteus • Thermotoga neapolitana • Thermus aquaticus • Treponema denticola • Trichomonas vaginalis • Tritrichomonas suis • Vibrionaceae • Xanthomonas axonopodis • Xanthomonas campestris • Xylella fastidiosa |
| L-histidine | C6H9N3O2 | • Osmoprotectant | • Arabidopsis thaliana • Bacillus subtilis • Brassica oleracea • Cavia porcellus • Clostridium perfringens • Clostridium tetanomorphum • Comamonas testosteroni • Delftia acidovorans • Drosophila  • Enterobacter aerogenes • Escherichia coli • Haemophilus influenzae • Homo sapiens • Klebsiella pneumoniae • Lactobacillus sp. 30A • Mammalia • Morganella morganii • Mus musculus • Mycobacterium avium • Mycobacterium smegmatis • Neurospora crassa • Oenococcus oeni • Pediococcus parvulus • Photobacterium damselae • Pseudomonas aeruginosa • Pseudomonas fluorescens • Pseudomonas putida • Raoultella planticola • Rattus norvegicus • Saccharomyces cerevisiae • Salmonella enterica • Staphylococcus capitis • Tetragenococcus muriaticus • Thermococcus onnurineus • Thermotoga maritima • Thermus thermophilus |
| L-methionine | C5H11N1O2S1 | • Osmoprotectant | • Acidothermus cellulolyticus • Aliivibrio fischeri • Anabaena variabilis • Aphanothece halophytica • Aquifex aeolicus • Arabidopsis thaliana • Archaeoglobus fulgidus • Arthrobacter • Bacillus circulans • Bacillus halodurans • Bacillus subtilis • Blautia producta • Bradyrhizobium japonicum • [Brevibacterium] flavum • Camellia sinensis • Campylobacter jejuni • Catharanthus roseus • Chlamydia muridarum • Chlamydia trachomatis • Chlamydophila abortus • Chlamydophila caviae • Chlamydophila felis • Citrobacter intermedius • Cladosporium cladosporioides • Clostridium botulinum • Clostridium formicaceticum • Corynebacterium glutamicum • Corynebacterium sp. • Cryptococcus albidus • Cucumis melo • Cupriavidus necator • Deinococcus radiodurans • Dianthus caryophyllus • Emiliania huxleyi • Entamoeba histolytica • Escherichia coli • Glycine max • Halobacteria • Halorhodospira halophila • Helicobacter pylori • Homo sapiens • Hordeum vulgare • Hyphomicrobium methylovorum • Hyphomicrobium zavarzinii • Klebsiella oxytoca • Klebsiella pneumoniae • Lactobacillus casei • Lactococcus lactis • Leptospira borgpetersenii • Leptospira interrogans • Leptospira meyeri • Lupinus luteus • Malus domestica • Melosira nummuloides • Mesorhizobium loti • Methanobacteria • Methanobrevibacter • Methanocaldococcus jannaschii • Methanococci • Methanoculleus • Methanogenium • Methanomicrobia • Methanosarcina • Methanosarcina thermophila • Methanothermobacter thermautotrophicus • Methanothermus • Methylobacterium extorquens • Methylobacterium organophilum • Micromonospora megalomicea • Moorella thermoacetica • Musca domestica • Mycobacterium • Mycobacterium bovis • Mycobacterium smegmatis • Mycobacterium tuberculosis • Nitratiruptor sp. SB155-2 • Nitrosococcus oceani • Nitrosospira multiformis • Nocardia • Oryza • Oryza sativa • Penicillium digitatum • Penicillium janthinellum • Pisum sativum • Plantago major • Pseudomonas aeruginosa • Pseudomonas putida • Pseudomonas sp. MS • Pyrobaculum calidifontis • Pyrococcus abyssi • Pyrococcus horikoshii • Ralstonia solanacearum • Rattus • Rattus norvegicus • Rhodopseudomonas palustris • Rhodospirillum rubrum • Saccharomyces cerevisiae • Saccharopolyspora erythraea • Salinispora tropica • Salmonella enterica • Schizosaccharomyces pombe • Sinorhizobium meliloti Rm2011 • Solanum lycopersicum • Solanum tuberosum • Spartina alterniflora • Spinacia oleracea • Staphylococcus aureus • Staphylococcus sciuri • Staphylococcus warneri • Streptococcus sanguinis • Streptomyces • Streptomyces aureofaciens • Streptomyces avermitilis • Streptomyces cattleya • Streptomyces coelicolor • Streptomyces venezuelae • Synechocystis • Synechocystis sp. PCC 6803 • Tetrahymena thermophila • Tetraselmis • Thermus thermophilus • Triticum • Triticum aestivum • Ulva intestinalis • Ulva lactuca • Vigna radiata • Wedelia biflora • Zea mays |
| glycine | C2H5N1O2 | • Osmoprotectant | • Achromobacter denitrificans • Achromobacter xylosoxidans  • Actinopolyspora halophila • Aeromonas jandaei • Aminobacter aminovorans • Aphanothece halophytica • Arabella iricolor • Arabidopsis thaliana • Archaeoglobus fulgidus • Arthrobacter • Arthrobacter sp. • Arthrobacter sp. TE1826 • Aves • Bacillus anthracis • Bacillus cereus • Bacillus subtilis • Bacillus thuringiensis • Bacteroides fragilis • Bifidobacterium longum • Blautia producta • Bos taurus • Brassica juncea • Busycotypus canaliculatus • Candida humicola • Cavia porcellus • Cellana grata • Chelatococcus asaccharovorans • Clostridium • Clostridium acidurici • Clostridium cylindrosporum • Clostridium formicaceticum • Clostridium innocuum • Clostridium paraputrificum • Clostridium pasteurianum • Clostridium perfringens • Clostridium purinilyticum • Clostridium sp. ATCC 29733 • [Clostridium] sticklandii • Corynebacterium sp. • Crassostrea gigas • Cryptococcus albidus • Emiliania huxleyi • Escherichia coli • Euglena gracilis • Gallus gallus • Glycine max • Halichondria japonica • Haliotis tuberculata • Halorhodospira halochloris • Homo sapiens • Hordeum vulgare • Hyphomicrobium methylovorum • Hyphomicrobium zavarzinii • Klebsiella oxytoca • Klebsiella pneumoniae • Kluyveromyces marxianus • Lactobacillus casei • Littorina littorea • Lotus japonicus • Lupinus luteus • Macaca fuscata • Macaca mulatta • Mammalia • Marphysa sanguinea • Melosira nummuloides • Meretrix lusoria • Methanobacteria • Methanobrevibacter • Methanocaldococcus jannaschii • Methanococci • Methanoculleus • Methanogenium • Methanohalophilus portucalensis • Methanosarcina • Methanothermobacter thermautotrophicus • Methanothermus • Methylobacterium extorquens • Methylobacterium organophilum • Methylobacter whittenburyi • Methylocystis echinoides • Methylocystis minimus • Methylocystis parvus • Methylocystis pyriformis • Methylosinus sporium • Methylosinus trichosporium • Moorella thermoacetica • Musca domestica • Mus musculus • Neurospora crassa • Oryctolagus cuniculus • Oryza sativa • Paracoccus denitrificans • Patiria pectinifera • Pecten jacobaeus • Pecten maximus • Penicillium digitatum • Pisum sativum • Plantago major • Plasmodium falciparum • Populus tremula x Populus alba • Pseudomonas aeruginosa • Pseudomonas cruciviae • Pseudomonas putida • Pseudomonas sp. MS • Pseudomonas sp. NCIMB 10558 • Pseudomonas sp. Ps7 • Pteris vittata • Pyrococcus furiosus • Pyrococcus horikoshii • Ralstonia solanacearum • Rattus • Rattus norvegicus • Rhodobacter capsulatus • Rhodobacter sphaeroides • Saccharomyces cerevisiae • Salmonella enterica • Scapharca broughtonii • Schizosaccharomyces pombe • Sinorhizobium meliloti Rm2011 • Solanum lycopersicum • Solanum tuberosum • Spinacia oleracea • Suberites domuncula • Sus scrofa • Tetraselmis • Tissierella creatinini • Tissierella creatinophila • Triticum aestivum • Ulva intestinalis • Ulva lactuca • uncultured bacterium HF130_AEPn_1 • Wedelia biflora • Xanthomonas campestris |
| L-alanine | C3H7N1O2 | • Osmoprotectant | • Acinetobacter • Aerococcus viridans • Amaranthus cruentus • Amaranthus hypochondriacus • Anabaena sp. 90 • Arabella iricolor • Arabidopsis thaliana • Archaeoglobus fulgidus • Arenicola marina • Arthrobacter aurescens • Arthrobacter citreus • Arthrobacter crystallopoietes • Arthrobacter polychromogenes • Arthrobacter sp. • Ascaris lumbricoides • Ascaris suum • Bacillus anthracis • Bacillus cereus • Bacillus megaterium • Bacillus subtilis • Bilophila wadsworthia • Blighia sapida • Bordetella pertussis • Burkholderia cenocepacia • Burkholderia cepacia • Burkholderia xenovorans • Busycotypus canaliculatus • Candida maltosa • Canis lupus • Carthamus tinctorius • Castellaniella denitrificans • Cellana grata • Claviceps purpurea • Clostridium propionicum • Conium maculatum • Corbicula japonica • Crassostrea gigas • Cryptococcus albidus • Cupriavidus metallidurans • Delftia acidovorans • Desulfonispora thiosulfatigenes • Emiliania huxleyi • Enterobacter aerogenes • Enterococcus faecalis • Epichloe festucae • Escherichia coli • Fasciola hepatica • Felis catus • Francisella novicida • Glycine max • Haemophilus influenzae • Halichondria japonica • Haliotis tuberculata • Homo sapiens • Hordeum vulgare • Klebsiella oxytoca • Klebsiella pneumoniae • Kocuria rosea • Lactobacillus salivarius • Lactococcus lactis • Leuconostoc mesenteroides • Littorina littorea • Lupinus albus • Lupinus luteus • Lupinus polyphyllus • Lupinus termis • Lysinibacillus sphaericus • Marphysa sanguinea • Megathyrsus maximus • Melosira nummuloides • Meretrix lusoria • Mesorhizobium loti • Methanobacteria • Methanobrevibacter • Methanocaldococcus jannaschii • Methanococci • Methanoculleus • Methanogenium • Methanosarcina • Methanothermobacter thermautotrophicus • Methanothermus • Methylobacillus flagellatus • Meyerozyma guilliermondii • Micrococcus luteus • Musca domestica • Mus musculus • Mytilus californianus • Mytilus edulis • Mytilus galloprovincialis • Mytilus trossulus • Nostoc punctiforme • Ochrobactrum • Oryza sativa • Panicum miliaceum • Paracoccus denitrificans • Patiria pectinifera • Pecten jacobaeus • Pecten maximus • Penicillium digitatum • Phaseolus vulgaris • Phormidium lapideum • Pisum sativum • Plantago major • Prochlorococcus marinus • Pseudomonas aeruginosa • Pseudomonas chlororaphis • Pseudomonas fluorescens • Pseudomonas mendocina • Pseudomonas sp. KIE171 • Pseudomonas sp. MA-1 • Ralstonia solanacearum • Rattus • Rattus norvegicus • Rhodococcus opacus • Saccharomyces cerevisiae • Saccharum officinarum • Salmonella enterica • Scapharca broughtonii • Schizosaccharomyces pombe • Sinorhizobium meliloti • Sipunculus nudus • Solanum lycopersicum • Solanum tuberosum • Sporosarcina pasteurii • Sporosarcina ureae • Staphylococcus aureus • Staphylococcus epidermidis • Staphylococcus haemolyticus • Streptococcus equinus • Streptococcus pasteurianus • Streptococcus thermophilus • Streptomyces bikiniensis • Streptomyces galbus • Streptomyces griseus • Streptomyces hygroscopicus • Streptomyces ornatus • Streptomyces parvulus • Streptomyces viridochromogenes • Suberites domuncula • Tetraselmis • Thermobifida fusca • Thermus thermophilus • Triticum aestivum • Ulva intestinalis • Ulva lactuca • Urochloa panicoides • Wedelia biflora • Weissella viridescens • Xanthobacter • Xanthomonas arboricola • Zea mays |
| L-lysine | C6H15N2O2 | • Osmoprotectant • Precursor | • Acetohalobium arabaticum • Aerococcus viridans • Aeropyrum pernix • Agrobacterium tumefaciens • Aliivibrio salmonicida • Anaerovibrio lipolyticus • Arabidopsis thaliana • Archaeoglobus fulgidus • Arthrobacter aurescens • Arthrobacter citreus • Arthrobacter crystallopoietes • Arthrobacter globiformis • Arthrobacter polychromogenes • Azotobacter vinelandii • Bacillus cereus • Bacillus circulans • Bacillus licheniformis • Bacillus megaterium • Bacillus mycoides • Bacillus pumilus • Bacillus subtilis • Bilophila wadsworthia • Bordetella pertussis • Bos taurus • Brevibacillus laterosporus • Brevibacterium • Candida maltosa L4 • Candida tropicalis • Chlamydia trachomatis • Clostridium peptidivorans • Clostridium SB4  • [Clostridium] sticklandii • Clostridium subterminale • Corynebacterium glutamicum • Cryptococcus albidus • Cyberlindnera saturnus • Deinococcus radiodurans • Desulfitobacterium hafniense • Emiliania huxleyi • Enterobacter aerogenes • Enterococcus faecalis • Escherichia coli • Euglena gracilis • [Flavobacterium] lutescens • Fusobacterium nucleatum • Geobacillus stearothermophilus • Glycine max • Haemophilus influenzae • Helicobacter pylori • Homo sapiens • Hordeum vulgare • Klebsiella oxytoca • Klebsiella pneumoniae • Kocuria rosea • Lactobacillus salivarius • Lactococcus lactis • Leuconostoc mesenteroides • Lupinus albus • Lupinus luteus • Lupinus polyphyllus • Lupinus termis • Lysinibacillus sphaericus • Melosira nummuloides • Methanobacteria • Methanobrevibacter • Methanocaldococcus jannaschii • Methanococci • Methanococcoides burtonii • Methanoculleus • Methanogenium • Methanohalobium evestigatum • Methanohalophilus mahii • Methanosarcina • Methanosarcina acetivorans • Methanosarcina barkeri • Methanosarcina thermophila • Methanothermobacter thermautotrophicus • Methanothermus • Meyerozyma guilliermondii • Micrococcus luteus • Musca domestica • Mus musculus • Mycobacterium tuberculosis • Neurospora crassa 15069 • Neurospora crassa 33933 • Nicotiana tabacum • Oryza sativa • Paenibacillus macerans • Paenibacillus polymyxa • Penicillium digitatum • Pisum sativum • Plantago major • Porphyromonas gingivalis • Proteus vulgaris • Pseudoalteromonas haloplanktis • Pseudomonas aeruginosa • Pseudomonas fluorescens • Pseudomonas putida • Pyrococcus abyssi • Pyrococcus horikoshii • Ralstonia solanacearum • Rattus • Rattus norvegicus • Rhizoctonia leguminicola • Rhodospirillum rubrum • Saccharomyces cerevisiae • Salmonella enterica • Schizosaccharomyces pombe • Selenomonas ruminantium • Shigella boydii • Shigella flexneri • Solanum lycopersicum • Solanum tuberosum • Sporosarcina globispora • Sporosarcina pasteurii • Sporosarcina ureae • Staphylococcus aureus • Staphylococcus epidermidis • Staphylococcus haemolyticus • Streptococcus equinus • Streptococcus pasteurianus • Streptococcus thermophilus • Streptomyces clavuligerus • Streptomyces coelicolor • Streptomyces pilosus • Sulfolobus acidocaldarius • Sulfolobus solfataricus • Sulfolobus tokodaii • Synechocystis • Tetraselmis • Thermincola potens • Thermus thermophilus • Trichoderma harzianum • Trichoderma viride • Ulva intestinalis • Ulva lactuca • Veillonella parvula • Virgibacillus pantothenticus • Wedelia biflora • Weissella viridescens • Yarrowia lipolytica • Yersinia frederiksenii • Yersinia intermedia • Yersinia kristensenii • Zea mays |
| L-proline | C5H9N1O2 | • Osmoprotectant | • Agrobacterium tumefaciens • Alkaliphilus metalliredigens • Alkaliphilus oremlandii • Aneurinibacillus migulanus • Arabidopsis thaliana • Archaeoglobus fulgidus • Bacillus brevis • Bacillus subtilis • Bos taurus • Bradyrhizobium japonicum • Brucella melitensis • Caldanaerobacter subterraneus • Claviceps purpurea • [Clostridium] difficile • Clostridium sporogenes • [Clostridium] sticklandii • Cryptococcus albidus • Datura stramonium • Emiliania huxleyi • Epichloe festucae • Escherichia coli • Fervidobacterium nodosum • Glycine max • Homo sapiens • Hordeum vulgare • Klebsiella oxytoca • Klebsiella pneumoniae • Lupinus angustifolius • Lupinus luteus • Melosira nummuloides • Methanobacteria • Methanobrevibacter • Methanocaldococcus jannaschii • Methanococci • Methanoculleus • Methanogenium • Methanosarcina • Methanothermobacter thermautotrophicus • Methanothermus • Musca domestica • Mus musculus • Natranaerobius thermophilus • Nicotiana tabacum • Oryza sativa • Pectobacterium carotovorum • Penicillium digitatum • Petrotoga mobilis • Photobacterium leiognathi • Photorhabdus luminescens • Pisum sativum • Plantago major • Propionibacterium acnes • Pseudomonas putida • Ralstonia solanacearum • Rattus • Rattus norvegicus • Rhizobiaceae • Rhodobacter capsulatus • Saccharomyces cerevisiae • Salmonella enterica • Schizosaccharomyces pombe • Sinorhizobium meliloti Rm2011 • Solanum lycopersicum • Solanum tuberosum • Synechocystis sp. PCC 6803 • Tetrahymena pyriformis • Tetraselmis • Thermoanaerobacter pseudethanolicus • Thermosipho melanesiensis • Ulva intestinalis • Ulva lactuca • Vigna aconitifolia • Wedelia biflora |
| L-asparagine | C4H8N2O3 | • Osmoprotectant | • Arabidopsis thaliana • Archaeoglobus fulgidus • Bacillus subtilis • Bos taurus • Cryptococcus albidus • Cylindrocarpon obtusisporum • Deinococcus radiodurans • Emiliania huxleyi • Erwinia chrysanthemi • Escherichia coli • Glycine max • Homo sapiens • Hordeum vulgare • Klebsiella oxytoca • Klebsiella pneumoniae • Linum usitatissimum • Lupinus albus • Lupinus angustifolius • Lupinus luteus • Malus domestica • Melosira nummuloides • Methanobacteria • Methanobrevibacter • Methanocaldococcus jannaschii • Methanococci • Methanoculleus • Methanogenium • Methanosarcina • Methanothermobacter thermautotrophicus • Methanothermus • Musca domestica • Nicotiana tabacum • Oryza sativa • Pectobacterium carotovorum • Penicillium digitatum • Pisum sativum • Plantago major • Ralstonia solanacearum • Rattus • Rattus norvegicus • Saccharomyces cerevisiae • Schizosaccharomyces pombe • Solanum lycopersicum • Solanum tuberosum • Sorghum bicolor • Spinacia oleracea • Tetraselmis • Thermus thermophilus • Ulva intestinalis • Ulva lactuca • Vicia faba • Wedelia biflora • Zea mays |
| L-glutamate | C5H8N1O4 | • Osmoprotectant | • Acer pseudoplatanus • Achromobacter superficialis • Acidaminococcus fermentans • Acidianus ambivalens • Acidithiobacillus thiooxidans • Acinetobacter lwoffii • Acinetobacter sp. NCIMB9871 • Acinetobacter sp. YAA • Actinobacteria  • Actinoplanes missouriensis • Aedes aegypti • Aerococcus viridans • Aeromonas caviae • Aeropyrum pernix • Agrobacterium tumefaciens • Alcaligenes faecalis • Alkaliphilus metalliredigens • Alkaliphilus oremlandii • Allium cepa • Allochromatium vinosum • Amaranthus cruentus • Amaranthus hypochondriacus • Aminobacter aminovorans • Amycolatopsis mediterranei • Amycolatopsis orientalis • Anaeromusa acidaminophila • Anchusa officinalis • Aneurinibacillus thermoaerophilus • Aquifex aeolicus • Arabidopsis thaliana • Archaeoglobus fulgidus • Arenicola marina • Arthrobacter aurescens • Arthrobacter citreus • Arthrobacter crystallopoietes • Arthrobacter globiformis • Arthrobacter nicotinovorans • Arthrobacter polychromogenes • Arthrobacter sp. KI72 • Ascaris lumbricoides • Ascaris suum • Aspergillus fumigatus • Aspergillus nidulans • Azospirillum brasilense • Azospirillum lipoferum • Azotobacter vinelandii • Bacillus caldovelox • Bacillus cereus • Bacillus circulans • Bacillus licheniformis • Bacillus megaterium • Bacillus mycoides • Bacillus pumilus • Bacillus sp. 8 • Bacillus subtilis • Bacteroides fragilis • Bacteroides thetaiotaomicron • Berberis stolonifera • Bordetella pertussis • Bos taurus • Bradyrhizobium japonicum • Brassica juncea • Brassica napus • Brassica oleracea • Brevibacillus laterosporus • Brevibacterium helvolum • Brucella abortus • Brucella melitensis • Burkholderia cepacia • Burkholderia lata • Burkholderia pyrrocinia • Burkholderia sp. JS667 • Burkholderia thailandensis • Caldanaerobacter subterraneus • Campylobacter coli • Campylobacter jejuni • Candida albicans • Candida maltosa • Candida maltosa L4 • Candida tropicalis • Canis lupus • Cavia porcellus • Chlamydia trachomatis • Chlamydomonas • Chlorobaculum tepidum • Chromatium • Chromobacterium violaceum • Chromohalobacter salexigens • Citrobacter amalonaticus • Citrobacter freundii • Clostridium • Clostridium aminobutyricum • Clostridium botulinum • Clostridium cochlearium • [Clostridium] difficile • Clostridium limosum • Clostridium malenominatum • Clostridium pasteurianum • Clostridium propionicum • Clostridium saccharobutylicum • Clostridium sporogenes • Clostridium sporosphaeroides • [Clostridium] sticklandii • Clostridium symbiosum • Clostridium tetani • Clostridium tetanomorphum • Comamonas testosteroni • Coptis japonica • Corbicula japonica • Corynebacterium glutamicum • Corynebacterium sp. • Crassostrea gigas • Cryptococcus albidus • Cucumis melo • Cupriavidus metallidurans • Cupriavidus necator • Cyanobium gracile • Cyberlindnera jadinii • Cyberlindnera saturnus • Cytophaga hutchinsonii • Datura stramonium • Deinococcus radiodurans • Delftia acidovorans • Delftia sp. AN3 • Delftia tsuruhatensis • Desulfovibrio desulfuricans • Desulfovibrio gigas • Desulfovibrio multispirans • Desulfovibrio vulgaris • Drosophila melanogaster • Emiliania huxleyi • Enterobacter aerogenes • Enterobacter cloacae • Enterococcus faecalis • Escherichia coli • Escherichia coli Crookes • Escherichia coli K4 • Eschscholzia californica • Euglena gracilis • Fasciola hepatica • Felis catus • Fervidobacterium nodosum • [Flavobacterium] lutescens • Frateuria sp. ANA-18 • Fusobacterium nucleatum • Geminocystis herdmanii • Gemmata sp. Wa1-1 • Geobacillus stearothermophilus • Glycine max • Gossypium hirsutum • Haemophilus influenzae • Halalkalicoccus • Haloarcula • Haloarcula marismortui • Halobacterium salinarum • Haloferax • Haloferax mediterranei • Haloferax volcanii • Halomicrobium • Haloterrigena • Helicobacter pylori • Homo sapiens • Hordeum vulgare • Hyperthermus butylicus • Ignicoccus • Ignicoccus hospitalis • Klebsiella oxytoca • Klebsiella pneumoniae • Kocuria rosea • Lactobacillaceae • Lactobacillus casei • Lactobacillus helveticus • Lactobacillus salivarius • Lactococcus lactis • Legionella pneumophila • Leptospira biflexa • Leptospira interrogans • Leuconostoc mesenteroides • Lithospermum erythrorhizon • Lotus japonicus • Lupinus angustifolius • Lupinus luteus • Lysinibacillus sphaericus • Macaca fuscata • Macaca mulatta • Mammalia • Megathyrsus maximus • Melissa officinalis • Melosira nummuloides • Metallosphaera sedula • Methanobacteria • Methanobacterium bryantii • Methanobrevibacter • Methanobrevibacter arboriphilus • Methanobrevibacter ruminantium • Methanobrevibacter smithii • Methanocaldococcus jannaschii • Methanococci • Methanococcus • Methanococcus aeolicus • Methanococcus maripaludis • Methanococcus voltae • Methanoculleus • Methanogenium • Methanomicrobia • Methanopyri • Methanosarcina • Methanosarcina acetivorans • Methanosarcina barkeri • Methanospirillum hungatei • Methanothermobacter thermautotrophicus • Methanothermus • Methylocella silvestris • Methyloversatilis universalis • Methylovorus mays • Micrococcus luteus • Micromonospora echinospora • Micromonospora megalomicea • Morganella morganii • Moritella sp. • Musca domestica • Mus musculus • Mycobacterium • Mycobacterium avium • Mycobacterium bovis • Mycobacterium smegmatis • Mycobacterium tuberculosis • Mytilus californianus • Mytilus edulis • Mytilus galloprovincialis • Mytilus trossulus • Myxococcus xanthus • Natranaerobius thermophilus • Natrialba • Natrialba magadii • Natronomonas • Neisseriaceae • Neisseria gonorrhoeae • Neisseria meningitidis • Neurospora crassa • Neurospora crassa 15069 • Neurospora crassa 33933 • Nicotiana rustica • Nicotiana sylvestris • Nicotiana tabacum • Nocardioides sp. JS614 • Oceanimonas doudoroffii • Oryza • Oryza sativa • Paenibacillus macerans • Paenibacillus polymyxa • Panicum miliaceum • Pantoea agglomerans • Papaver somniferum • Paracoccus pantotrophus NKNCYSA • Paracoccus thiophilus • Penicillium digitatum • Peptoniphilus asaccharolyticus • Petrotoga mobilis • Petunia x hybrida • Photobacterium leiognathi • Pimelobacter simplex • Pinus sylvestris • Pisum sativum • Plantago major • Polaribacter filamentus • Populus tremula x Populus alba • Portulaca grandiflora • Prevotella ruminicola • Propionibacterium acnes • Propionibacterium freudenreichii • Providencia alcalifaciens • Pseudoalteromonas haloplanktis • Pseudoalteromonas tetraodonis • Pseudomonas • Pseudomonas aeruginosa • Pseudomonas chlororaphis • Pseudomonas denitrificans • Pseudomonas fluorescens • Pseudomonas fluorescens 2-79 • Pseudomonas mendocina • Pseudomonas mevalonii • Pseudomonas oleovorans • Pseudomonas putida • Pseudomonas sp. KIE171 • Pseudomonas sp. P.J. 874 • Pseudomonas syringae • Pyrobaculum • Pyrococcus abyssi • Pyrococcus horikoshii • Ralstonia eutropha • Ralstonia solanacearum • Ralstonia sp. JS668 • Rattus • Rattus norvegicus • Rhizoctonia leguminicola • Rhodobacter capsulatus • Rhodococcus opacus • Rhodococcus rhodochrous • Rhodopseudomonas palustris • Rhodospirillum rubrum • Ricinus communis • Roseovarius nubinhibens • Ruegeria pomeroyi • Ruminococcus albus • Ruta graveolens • Saccharomyces cerevisiae • Saccharopolyspora • Saccharopolyspora erythraea • Saccharopolyspora spinosa • Salmonella enterica • Schizosaccharomyces pombe • Sinorhizobium meliloti • Sinorhizobium meliloti Rm2011 • Sipunculus nudus • Solanum lycopersicum • Solanum tuberosum • Solenostemon scutellarioides • Sorghum bicolor • Sphaerobacter • Spinacia oleracea • Sporosarcina pasteurii • Sporosarcina ureae • Staphylococcus aureus • Staphylococcus epidermidis • Staphylococcus haemolyticus • Streptococcus equinus • Streptococcus pasteurianus • Streptococcus thermophilus • Streptomyces • Streptomyces antibioticus • Streptomyces avermitilis • Streptomyces chrestomyceticus • Streptomyces clavuligerus • Streptomyces coelicolor • Streptomyces fradiae • Streptomyces griseus • Streptomyces kanamyceticus • Streptomyces lividus • Streptomyces luridus • Streptomyces mycarofaciens • Streptomyces ribosidificus • Streptomyces venezuelae • Sulfolobus acidocaldarius • Sulfolobus solfataricus • Sulfolobus tokodaii • Sus scrofa • Synechococcus elongatus • Synechocystis • Synechocystis sp. PCC 6803 • Tannerella forsythia • Tetrahymena pyriformis • Tetraselmis • Thalictrum flavum • Thalictrum tuberosum • Thauera aromatica • Thermoanaerobacterium thermosaccharolyticum • Thermoanaerobacter pseudethanolicus • Thermobaculum • Thermococcus kodakarensis • Thermococcus onnurineus • Thermomicrobium • Thermoproteus • Thermosipho melanesiensis • Thermotoga maritima • Thermotoga neapolitana • Thermus aquaticus • Thermus thermophilus • Thiobacillus thioparus • Triticum • Triticum aestivum • Ulva intestinalis • Ulva lactuca • Urochloa panicoides • Vibrio alginolyticus • Vibrio cholerae • Vibrionaceae • Vibrio parahaemolyticus • Vibrio vulnificus • Vigna aconitifolia • Virgibacillus pantothenticus • Vitis vinifera • Vogesella indigofera • Wedelia biflora • Weissella viridescens • Xanthomonas arboricola • Xanthomonas axonopodis • Xanthomonas campestris • Xylella fastidiosa • Yarrowia lipolytica • Yersinia pseudotuberculosis • Zea mays |
| L-isoleucine | C6H13N1O2 | • Osmoprotectant | • Arabidopsis thaliana • Archaeoglobus fulgidus • Bacillus subtilis • Bacteroides fragilis • Bos taurus • Chromatium • Clostridium • Clostridium pasteurianum • Clostridium sporogenes • Cryptococcus albidus • Desulfovibrio desulfuricans • Emiliania huxleyi • Escherichia coli • Escherichia coli Crookes • Glycine max • Homo sapiens • Hordeum vulgare • Ignicoccus hospitalis • Klebsiella oxytoca • Klebsiella pneumoniae • Leptospira biflexa • Leptospira interrogans • Linum usitatissimum • Lotus japonicus • Lupinus luteus • Manihot esculenta • Melosira nummuloides • Methanobacteria • Methanobacterium bryantii • Methanobrevibacter • Methanobrevibacter arboriphilus • Methanobrevibacter ruminantium • Methanobrevibacter smithii • Methanocaldococcus jannaschii • Methanococci • Methanococcus aeolicus • Methanococcus voltae • Methanoculleus • Methanogenium • Methanosarcina • Methanosarcina barkeri • Methanospirillum hungatei • Methanothermobacter thermautotrophicus • Methanothermus • Musca domestica • Nicotiana attenuata • Oryza sativa • Penicillium digitatum • Pisum sativum • Plantago major • Prevotella ruminicola • Pseudomonas • Pseudomonas aeruginosa • Pseudomonas putida • Ralstonia solanacearum • Rattus • Rattus norvegicus • Saccharomyces cerevisiae • Salmonella enterica • Schizosaccharomyces pombe • Solanum lycopersicum • Solanum tuberosum • Streptomyces avermitilis • Streptomyces venezuelae • Sulfolobus solfataricus • Tetraselmis • Trifolium repens • Ulva intestinalis • Ulva lactuca • Wedelia biflora |
| L-leucine | C6H13N1O2 | • Osmoprotectant | • Acer pseudoplatanus • Aneurinibacillus migulanus • Arabidopsis thaliana • Archaeoglobus fulgidus • Bacillus brevis • Bacillus subtilis • Clostridium sporogenes • Cryptococcus albidus • Emiliania huxleyi • Escherichia coli • Glycine max • Homo sapiens • Hordeum vulgare • Klebsiella oxytoca • Klebsiella pneumoniae • Leptospira interrogans • Lupinus luteus • Melosira nummuloides • Methanobacteria • Methanobrevibacter • Methanocaldococcus jannaschii • Methanococci • Methanoculleus • Methanogenium • Methanosarcina • Methanothermobacter thermautotrophicus • Methanothermus • Musca domestica • Nicotiana attenuata • Oryza sativa • Paenibacillus polymyxa • Penicillium digitatum • Pisum sativum • Plantago major • Pseudomonas aeruginosa • Pseudomonas fluorescens • Pseudomonas mevalonii • Pseudomonas oleovorans • Pseudomonas putida • Ralstonia solanacearum • Rattus • Rattus norvegicus • Saccharomyces cerevisiae • Schizosaccharomyces pombe • Solanum lycopersicum • Solanum tuberosum • Streptomyces avermitilis • Tetraselmis • Ulva intestinalis • Ulva lactuca • Wedelia biflora |
| L-arginine | C6H15N4O2 | • Osmoprotectant | • Acinetobacter sp. ADP1 • Aeromonas caviae • Agrobacterium tumefaciens • Aphanocapsa • Arabella iricolor • Arabidopsis thaliana • Archaeoglobus fulgidus • Arthrobacter globiformis • Arthrobacter sp. KUJ8602 • Avena sativa • Bacillus caldovelox • Bacillus licheniformis • Bacillus subtilis • Bacteroides thetaiotaomicron • Bos taurus • Brassica juncea • Brevibacterium helvolum • Brucella melitensis • Burkholderia cepacia • Busycotypus canaliculatus • Campylobacter jejuni • Capsicum annuum • Cellana grata • Chlamydomonas • [Clostridium] sticklandii • Corynebacterium glutamicum • Crassostrea gigas • Cryptococcus albidus • Cytophaga hutchinsonii • Datura stramonium • Dianthus caryophyllus • Emiliania huxleyi • Enterobacter aerogenes • Escherichia coli • Geminocystis herdmanii • Giardia intestinalis • Glycine max • Halalkalicoccus • Halichondria japonica • Haliotis tuberculata • Haloarcula • Halobacterium salinarum • Haloferax • Halomicrobium • Haloterrigena • Hexamita inflata • Homo sapiens • Hordeum vulgare • Ignicoccus • Klebsiella oxytoca • Klebsiella pneumoniae • Lactobacillus hilgardii • Leishmania donovani • Littorina littorea • Lupinus luteus • Malus domestica • Marphysa sanguinea • Melosira nummuloides • Meretrix lusoria • Metallosphaera sedula • Methanobacteria • Methanobrevibacter • Methanocaldococcus jannaschii • Methanococci • Methanoculleus • Methanogenium • Methanosarcina • Methanothermobacter thermautotrophicus • Methanothermus • Moritella sp. • Musca domestica • Mus musculus • Mycobacterium phlei • Mycobacterium smegmatis • Mycoplasma hominis • Mycoplasma pneumoniae • Myxococcus xanthus • Natrialba • Natronomonas • Neisseriaceae • Neurospora crassa • Nicotiana tabacum • Oceanimonas doudoroffii • Oryza sativa • Patiria pectinifera • Pecten jacobaeus • Pecten maximus • Penicillium digitatum • Pimelobacter simplex • Pinus sylvestris • Pisum sativum • Plantago major • Prevotella ruminicola • Pseudomonas • Pseudomonas aeruginosa • Pseudomonas chlororaphis • Pseudomonas fluorescens • Pseudomonas mendocina • Pseudomonas putida • Pseudomonas syringae • Pyrobaculum • Ralstonia solanacearum • Rattus • Rattus norvegicus • Rhizobiaceae • Saccharomyces cerevisiae • Salmonella enterica • Scapharca broughtonii • Schizosaccharomyces pombe • Sinorhizobium meliloti Rm2011 • Solanum lycopersicum • Solanum tuberosum • Sphaerobacter • Spiroplasma citri • Streptococcus ratti • Streptomyces bikiniensis • Streptomyces clavuligerus • Streptomyces galbus • Streptomyces garyphalus • Streptomyces griseus • Streptomyces lavendulae • Streptomyces ornatus • Streptomyces violaceochromogenes • Suberites domuncula • Sulfolobus acidocaldarius • Sulfolobus solfataricus • Synechocystis sp. PCC 6803 • Tannerella forsythia • Tetrahymena pyriformis • Tetraselmis • Theobroma cacao • Thermobaculum • Thermococcus kodakarensis • Thermomicrobium • Thermoproteus • Thermosynechococcus elongatus • Thermotoga neapolitana • Thermus aquaticus • Thermus thermophilus • Treponema denticola • Trichomonas vaginalis • Tritrichomonas suis • Ulva intestinalis • Ulva lactuca • Vibrionaceae • Vogesella indigofera • Wedelia biflora • Xanthomonas axonopodis • Xanthomonas campestris • Xylella fastidiosa |
| L-threonine | C4H9N1O3 | • Osmoprotectant | • Achromobacter xylosoxidans  • Aeromonas jandaei • Arabidopsis thaliana • Archaeoglobus fulgidus • Arthrobacter globiformis • Arthrobacter sp. • Aves • Bacillus anthracis • Bacillus cereus • Bacillus subtilis • Bacillus thuringiensis • Blighia sapida • Candida humicola • Clostridium pasteurianum • [Clostridium] sticklandii • Clostridium tetanomorphum • Corynebacterium sp. • Cryptococcus albidus • Emiliania huxleyi • Escherichia coli • Glycine max • Homo sapiens • Hordeum vulgare • Klebsiella oxytoca • Klebsiella pneumoniae • Lupinus luteus • Mammalia • Melosira nummuloides • Methanobacteria • Methanobrevibacter • Methanocaldococcus jannaschii • Methanococci • Methanococcus voltae • Methanoculleus • Methanogenium • Methanosarcina • Methanosarcina thermophila • Methanothermobacter thermautotrophicus • Methanothermus • Musca domestica • Oryza sativa • Paenibacillus polymyxa • Penicillium digitatum • Pisum sativum • Plantago major • Pseudomonas aeruginosa • Pseudomonas cruciviae • Pseudomonas denitrificans • Pseudomonas putida • Pseudomonas sp. NCIB 8858 • Pseudomonas sp. NCIMB 10558 • Pyrococcus furiosus • Pyrococcus horikoshii • Ralstonia solanacearum • Rattus • Rattus norvegicus • Saccharomyces cerevisiae • Salmonella enterica • Schizosaccharomyces pombe • Serratia marcescens • Solanum lycopersicum • Solanum tuberosum • Streptomyces cattleya • Tetraselmis • Ulva intestinalis • Ulva lactuca • Vibrio cholerae • Wedelia biflora • Xanthomonas campestris |
| L-glutamine | C5H10N2O3 | • Osmoprotectant | • Acidianus ambivalens • Actinobacteria  • Aedes aegypti • Allium cepa • Allochromatium vinosum • Amycolatopsis mediterranei • Aquifex aeolicus • Arabidopsis thaliana • Archaeoglobus fulgidus • Azospirillum brasilense • Bacillus cereus • Bacillus circulans • Bacillus licheniformis • Bacillus megaterium • Bacillus pumilus • Bacillus subtilis • Bacteroides thetaiotaomicron • Bos taurus • Brassica oleracea • Brucella melitensis • Burkholderia lata • Burkholderia thailandensis • Campylobacter jejuni • Candida albicans • Cavia porcellus • Chlamydomonas • Chlorobaculum tepidum • Corynebacterium glutamicum • Cryptococcus albidus • Cupriavidus metallidurans • Cytophaga hutchinsonii • Deinococcus radiodurans • Desulfovibrio desulfuricans • Desulfovibrio gigas • Desulfovibrio multispirans • Desulfovibrio vulgaris • Drosophila melanogaster • Emiliania huxleyi • Escherichia coli • Gemmata sp. Wa1-1 • Geobacillus stearothermophilus • Glycine max • Gossypium hirsutum • Haemophilus influenzae • Halalkalicoccus • Haloarcula • Haloferax • Haloferax mediterranei • Haloferax volcanii • Halomicrobium • Haloterrigena • Helicobacter pylori • Homo sapiens • Hordeum vulgare • Hyperthermus butylicus • Ignicoccus • Ignicoccus hospitalis • Klebsiella oxytoca • Klebsiella pneumoniae • Leptospira interrogans • Lupinus angustifolius • Lupinus luteus • Macaca fuscata • Macaca mulatta • Melosira nummuloides • Metallosphaera sedula • Methanobacteria • Methanobrevibacter • Methanocaldococcus jannaschii • Methanococci • Methanoculleus • Methanogenium • Methanosarcina • Methanothermobacter thermautotrophicus • Methanothermus • Moritella sp. • Musca domestica • Mus musculus • Mycobacterium tuberculosis • Myxococcus xanthus • Natrialba • Natronomonas • Neisseriaceae • Neurospora crassa • Nicotiana attenuata • Nicotiana rustica • Nicotiana tabacum • Oryza sativa • Penicillium digitatum • Pisum sativum • Plantago major • Polaribacter filamentus • Prevotella ruminicola • Propionibacterium freudenreichii • Pseudomonas • Pseudomonas aeruginosa • Pseudomonas chlororaphis • Pseudomonas denitrificans • Pseudomonas fluorescens • Pseudomonas fluorescens 2-79 • Pseudomonas putida • Pyrobaculum • Ralstonia solanacearum • Rattus • Rattus norvegicus • Rhodobacter capsulatus • Ricinus communis • Ruminococcus albus • Ruta graveolens • Saccharomyces cerevisiae • Salmonella enterica • Schizosaccharomyces pombe • Sinorhizobium meliloti • Solanum lycopersicum • Solanum tuberosum • Sphaerobacter • Sporosarcina pasteurii • Streptoalloteichus tenebrarius • Streptomyces antibioticus • Streptomyces bikiniensis • Streptomyces fradiae • Streptomyces galbus • Streptomyces griseus • Streptomyces kanamyceticus • Streptomyces ornatus • Sulfolobus acidocaldarius • Sulfolobus solfataricus • Sulfolobus tokodaii • Sus scrofa • Synechococcus elongatus • Synechocystis sp. PCC 6803 • Tannerella forsythia • Tetraselmis • Thermobaculum • Thermococcus kodakarensis • Thermococcus onnurineus • Thermomicrobium • Thermoproteus • Thermotoga maritima • Thermotoga neapolitana • Thermus aquaticus • Thermus thermophilus • Ulva intestinalis • Ulva lactuca • Vibrionaceae • Vitis vinifera • Wedelia biflora • Xanthomonas arboricola • Xanthomonas axonopodis • Xanthomonas campestris • Xylella fastidiosa • Zea mays |
| L-valine | C5H11N1O2 | • Osmoprotectant | • Acremonium chrysogenum • Amycolatopsis lactamdurans • Aneurinibacillus migulanus • Arabidopsis thaliana • Archaeoglobus fulgidus • Aspergillus nidulans • Bacillus brevis • Bacillus subtilis • Bos taurus • Burkholderia cepacia • Cryptococcus albidus • Cyberlindnera jadinii • Emiliania huxleyi • Escherichia coli • Glycine max • Homo sapiens • Hordeum vulgare • Klebsiella oxytoca • Klebsiella pneumoniae • Lactococcus lactis • Linum usitatissimum • Lotus japonicus • Lupinus luteus • Manihot esculenta • Melosira nummuloides • Methanobacteria • Methanobrevibacter • Methanocaldococcus jannaschii • Methanococci • Methanococcus • Methanococcus aeolicus • Methanococcus maripaludis • Methanococcus voltae • Methanoculleus • Methanogenium • Methanosarcina • Methanothermobacter thermautotrophicus • Methanothermus • Musca domestica • Neurospora crassa • Nicotiana attenuata • Oryza sativa • Penicillium chrysogenum • Penicillium digitatum • Pisum sativum • Plantago major • Pseudomonas aeruginosa • Pseudomonas fluorescens • Pseudomonas putida • Ralstonia solanacearum • Rattus • Rattus norvegicus • Saccharomyces cerevisiae • Schizosaccharomyces pombe • Solanum lycopersicum • Solanum tuberosum • Streptomyces avermitilis • Streptomyces clavuligerus • Streptomyces coelicolor • Sus scrofa • Tetrahymena pyriformis • Tetraselmis • Ulva intestinalis • Ulva lactuca • Wedelia biflora |
| 1-aminocyclopropane-1-carboxylate | C4H7N1O2 | • Non-Osmolyte • Osmoregulation | • Arabidopsis thaliana • Cucumis melo • Dianthus caryophyllus • Glycine max • Lupinus luteus • Malus domestica • Oryza sativa • Plantago major • Solanum lycopersicum • Vigna radiata |
| L-ornithine | C5H13N2O2 | • Precursor | • Acidithiobacillus ferrooxidans • Aeromonas caviae • Agrobacterium tumefaciens • Alkaliphilus metalliredigens • Alkaliphilus oremlandii • Aneurinibacillus migulanus • Aphanocapsa • Arabidopsis thaliana • Avena sativa • Bacillus brevis • Bacillus caldovelox • Bacillus licheniformis • Bacillus subtilis • Bordetella pertussis • Bos taurus • Brassica juncea • Brucella melitensis • Burkholderia cepacia • Caldanaerobacter subterraneus • Capsicum annuum • Chlamydomonas • [Clostridium] difficile • Clostridium sporogenes • [Clostridium] sticklandii • Corynebacterium glutamicum • Datura stramonium • Dianthus caryophyllus • Escherichia coli • Fervidobacterium nodosum • Flavobacterium johnsoniae • Giardia intestinalis • Glycine max • Halalkalicoccus • Haloarcula • Halobacterium salinarum • Haloferax • Halomicrobium • Haloterrigena • Hexamita inflata • Homo sapiens • Hordeum vulgare • Ignicoccus • Lactobacillus hilgardii • Lactobacillus plantarum • Lupinus angustifolius • Malus domestica • Metallosphaera sedula • Methanothermobacter thermautotrophicus • Moritella sp. • Mus musculus • Mycobacterium tuberculosis complex • Mycoplasma hominis • Mycoplasma pneumoniae • Myxococcus xanthus • Natranaerobius thermophilus • Natrialba • Natronomonas • Neisseriaceae • Neurospora crassa • Nicotiana tabacum • Oenococcus oeni • Oryza sativa • Petrotoga mobilis • Pinus sylvestris • Pisum sativum • Propionibacterium acnes • Pseudomonas • Pseudomonas aeruginosa • Pseudomonas fluorescens • Pseudomonas putida • Pseudomonas stutzeri • Pyrobaculum • Rattus norvegicus • Rhizobiaceae • Rhodobacter capsulatus • Saccharomyces cerevisiae • Salmonella enterica • Sinorhizobium meliloti • Sinorhizobium meliloti Rm2011 • Solanum lycopersicum • Sphaerobacter • Spiroplasma citri • Streptococcus ratti • Streptomyces bikiniensis • Streptomyces galbus • Streptomyces garyphalus • Streptomyces griseus • Streptomyces lavendulae • Streptomyces ornatus • Sulfolobus acidocaldarius • Sulfolobus solfataricus • Synechocystis sp. PCC 6803 • Tetrahymena pyriformis • Theobroma cacao • Thermoanaerobacter pseudethanolicus • Thermobaculum • Thermomicrobium • Thermoproteus • Thermosipho melanesiensis • Thermotoga neapolitana • Thermus aquaticus • Treponema denticola • Trichomonas vaginalis • Tritrichomonas suis • Vibrionaceae • Vigna aconitifolia |
| N6-acetyl-L-lysine | C8H16N2O3 | • Osmoprotectant | • Candida maltosa L4 • Candida tropicalis • Cyberlindnera saturnus • Neurospora crassa 15069 • Neurospora crassa 33933 • Rhizoctonia leguminicola • Saccharomyces cerevisiae • Yarrowia lipolytica |
| N-acetyl-L-aspartate | C6H7N1O5 | • Osmoprotectant |  |
| N-methyl-L-glutamate | C6H10N1O4 | • Osmoprotectant | • Aminobacter aminovorans • Methylocella silvestris • Methyloversatilis universalis • Methylovorus mays |
| D-octopine | C9H18N4O4 | • Osmoprotectant | • Agrobacterium tumefaciens • Arabella iricolor • Busycotypus canaliculatus • Cellana grata • Crassostrea gigas • Halichondria japonica • Haliotis tuberculata • Littorina littorea • Marphysa sanguinea • Meretrix lusoria • Patiria pectinifera • Pecten jacobaeus • Pecten maximus • Scapharca broughtonii • Suberites domuncula |
| sarcosine | C3H7N1O2 | • Osmoprotectant | • Achromobacter denitrificans • Actinopolyspora halophila • Aphanothece halophytica • Arthrobacter • Arthrobacter sp. • Arthrobacter sp. TE1826 • Corynebacterium sp. • Halorhodospira halochloris • Homo sapiens • Methanohalophilus portucalensis • Pseudomonas aeruginosa • Pseudomonas putida • Pseudomonas sp. Ps7 • Rattus norvegicus • Sinorhizobium meliloti Rm2011 • Tissierella creatinini • Tissierella creatinophila |
| S-methyl-L-methionine | C6H14N1O2S1 | • Osmoprotectant | • Arabidopsis thaliana • Homo sapiens • Rattus norvegicus • Spartina alterniflora • Wedelia biflora |
| D-alanine | C3H7N1O2 | • Osmoprotectant | • Aerococcus viridans • Alkaliphilus metalliredigens • Alkaliphilus oremlandii • Arenicola marina • Arthrobacter aurescens • Arthrobacter citreus • Arthrobacter crystallopoietes • Arthrobacter polychromogenes • Arthrobacter viscosus • Ascaris lumbricoides • Ascaris suum • Bacillus anthracis • Bacillus megaterium • Bacillus subtilis • Caldanaerobacter subterraneus • [Clostridium] difficile • Clostridium sporogenes • [Clostridium] sticklandii • Corbicula japonica • Corynebacterium ammoniagenes • Corynebacterium diphtheriae • Corynebacterium glutamicum • Corynebacterium pseudotuberculosis • Crassostrea gigas • Enterococcus casseliflavus • Enterococcus faecalis • Enterococcus faecium • Enterococcus gallinarum • Enterococcus hirae • Enterococcus raffinosus • Escherichia coli • Fasciola hepatica • Fervidobacterium nodosum • Geobacillus stearothermophilus • Kocuria rosea • Lactobacillus salivarius • Lactococcus lactis • Leuconostoc mesenteroides • Micrococcus luteus • Mycobacterium leprae • Mycobacterium smegmatis • Mycobacterium tuberculosis • Mytilus californianus • Mytilus edulis • Mytilus galloprovincialis • Mytilus trossulus • Natranaerobius thermophilus • Petrotoga mobilis • Propionibacterium acnes • Propionibacterium freudenreichii • Pseudomonas aeruginosa • Sinomonas albida • Sipunculus nudus • Sporosarcina pasteurii • Sporosarcina ureae • Staphylococcus aureus • Staphylococcus epidermidis • Staphylococcus haemolyticus • Streptococcus equinus • Streptococcus pasteurianus • Streptococcus thermophilus • Thermoanaerobacter pseudethanolicus • Thermosipho melanesiensis • Weissella viridescens |
| creatine | C4H9N3O2 | • Osmoprotectant | • Achromobacter denitrificans • Arthrobacter sp. TE1826 • Gallus gallus • Homo sapiens • Oryctolagus cuniculus • Pseudomonas putida • Pseudomonas sp. Ps7 • Rattus norvegicus • Tissierella creatinini • Tissierella creatinophila |
| beta-alanine | C3H7N1O2 | • Osmoprotectant | • Arabella iricolor • Arabidopsis thaliana • Brevibacillus agri • Busycotypus canaliculatus • Carthamus tinctorius • Cellana grata • Crassostrea gigas • Datura inoxia • Escherichia coli • Francisella tularensis • Glycine max • Halichondria japonica • Haliotis tuberculata • Hordeum vulgare • Lachancea kluyveri • Limonium latifolium • Littorina littorea • Lotus japonicus • Marphysa sanguinea • Meretrix lusoria • Patiria pectinifera • Pecten jacobaeus • Pecten maximus • Phaseolus vulgaris • Pisum sativum • Pseudomonas fluorescens • Rattus norvegicus • Saccharomyces cerevisiae • Scapharca broughtonii • Setaria italica • Solanum lycopersicum • Spinacia oleracea • Suberites domuncula • Thermococcus kodakarensis • Triticum aestivum • Zea mays |
| verbascose | C30H52O26 | • Osmoprotectant | • Ajuga reptans • Pisum sativum |
| raffinose | C18H32O16 | • Osmoprotectant | • Ajuga reptans • Arabidopsis thaliana • Cerastium arvense • Cucumis melo • Cucumis sativus • Cucurbita pepo • Glycine max • Lens culinaris • Phaseolus vulgaris • Pisum sativum • Solanum lycopersicum • Stellaria media • Vicia faba • Vigna angularis |
| melibiose | C12H22O11 | • Osmoprotectant | • Escherichia coli |
| beta-palatinose | C12H22O11 | • Osmoprotectant |  |
| beta-gentiobiose | C12H22O11 | • Osmoprotectant | • Hevea brasiliensis • Linum usitatissimum |
| beta-turanose | C12H22O11 | • Osmoprotectant |  |
| sucrose | C12H22O11 | • Osmoprotectant | • Agrobacterium tumefaciens • Ajuga reptans • Allium cepa • Arabidopsis thaliana • Avena sativa • Bifidobacterium adolescentis • Bifidobacterium animalis • Bifidobacterium bifidum • Bifidobacterium longum • Cerastium arvense • Cucumis melo • Cucumis sativus • Cucurbita pepo • Glycine max • Helianthus tuberosus • Homo sapiens • Hordeum vulgare • Lens culinaris • Malus domestica • Methylobacillus flagellatus • Nicotiana tabacum • Oryza sativa • Phaseolus vulgaris • Phleum pratense • Pisum sativum • Solanum lycopersicum • Solanum tuberosum • Spinacia oleracea • Stellaria media • Triticum aestivum • Vicia faba • Vigna angularis |
| beta-maltose | C12H22O11 | • Osmoprotectant |  |
| alpha-maltose | C12H22O11 | • Osmoprotectant |  |
| alpha,alpha-trehalose | C12H22O11 | • Osmoprotectant | • Agaricus bisporus • Apis mellifera • Arthrobacter sp. Q36 • Asanoa ferruginea • Azorhizobium caulinodans • Bradyrhizobium elkanii • Bradyrhizobium japonicum • Brevibacterium helvolum • Corynebacterium glutamicum • Escherichia coli • Euglena gracilis • Flammulina velutipes • Geobacillus stearothermophilus • Grifola frondosa • Halorhodospira halochloris • Helicoverpa armigera • Kocuria varians • Lentinus sajor-caju • Mycobacterium avium • Mycobacterium bovis • Mycobacterium leprae • Mycobacterium smegmatis • Mycobacterium tuberculosis • Phormia regina • Pichia fermentans • Pimelobacter sp. • Plesiomonas sp. Yoshida 95 • Pleurotus ostreatus • Pseudomonas putida • Pseudomonas stutzeri • Pyrococcus horikoshii • Rhizobiaceae • Rhizobium leguminosarum • Rhizobium tropici • Saccharomyces cerevisiae • Schizophyllum commune • Scytonema sp. • Sinorhizobium fredii • Sinorhizobium meliloti Rm2011 • Streptomyces aureofaciens • Streptomyces bikiniensis • Streptomyces griseus • Streptomyces hygroscopicus • Streptomyces rimosus • Sulfolobus acidocaldarius • Thermoanaerobacter brockii • Thermococcus litoralis • Thermoproteus tenax • Thermus aquaticus • Thermus caldophilus |
| stachyose | C24H42O21 | • Osmoprotectant | • Ajuga reptans • Arabidopsis thaliana • Cucumis melo • Cucumis sativus • Cucurbita pepo • Glycine max • Lens culinaris • Phaseolus vulgaris • Pisum sativum • Solanum lycopersicum • Vicia faba • Vigna angularis |
| cyclic-GMP | C10H11N5O7P1 | • Non-Osmolyte • Osmoregulation |  |
| 4-phenylbutyrate | C10H11O2 | • Osmoprotectant |  |
| flavone | C15H10O2 | • Osmoregulation |  |
| 3-hydroxyflavone | C15H9O3 | • Osmoregulation |  |
| genistein | C15H9O5 | • Osmoregulation | • Cicer arietinum • Dianthus caryophyllus • Gerbera hybrid cultivar • Glycine max • Glycyrrhiza echinata • Glycyrrhiza glabra • Lupinus albus • Medicago sativa • Medicago truncatula • Petroselinum crispum • Pueraria montana • Trifolium pratense |
| daidzein | C15H10O4 | • Osmoregulation | • Adlercreutzia equolifaciens • Asaccharobacter celatus • Cicer arietinum • Glycine max • Glycyrrhiza echinata • Glycyrrhiza glabra • human intestinal bacterium SNU-Julong732 • Lactococcus garvieae • Lotus japonicus • Medicago sativa • Medicago truncatula • Phaseolus vulgaris • Pueraria montana • Slackia isoflavoniconvertens • Slackia sp. NATTS • Trifolium pratense • Vigna radiata |
| O-acetylcarnitine | C9H17N1O4 | • Osmoprotectant | • Bos taurus • Homo sapiens • Mus musculus • Ovis aries • Rattus norvegicus • Sus scrofa |
| 2,3-diphospho-D-glycerate | C3H3O10P2 | • Osmoprotectant | • Dictyostelium discoideum • Homo sapiens |
| 2-O-alpha-mannosyl-D-glycerate | C9H15O9 | • Osmoprotectant | • Dehalococcoides mccartyi • Methanothermus fervidus • Petrotoga miotherma • Pyrococcus furiosus • Pyrococcus horikoshii • Rhodothermus marinus • Rubrobacter xylanophilus • Thermococcus celer • Thermococcus litoralis • Thermococcus stetteri • Thermus thermophilus |
| 2-[2-O-(alpha-D-mannopyranosyl)-alpha-D-glucopyranosyl]-3-phospho-D-glycerate | C15H24O17P1 | • Osmoprotectant | • Petrotoga miotherma • Petrotoga mobilis |
| 2-O-(alpha-D-glucopyranosyl)-D-glycerate | C9H15O9 | • Osmoprotectant | • Erwinia chrysanthemi • Halomonas elongata • Methanococcoides burtonii • Methanohalophilus portucalensis • Persephonella marina • Petrotoga miotherma • Petrotoga mobilis • Synechococcus sp. PCC 7002 • Thermotoga maritima |
| L-1-glycero-3-phosphocholine | C8H20N1O6P1 | • Osmoprotectant | • Deinococcus radiodurans • Escherichia coli • Saccharomyces cerevisiae • Synechocystis |
| androsterone | C19H30O2 | • Non-Osmolyte • Osmoregulation | • Aquilegia vulgaris • Arabidopsis thaliana • Comamonas testosteroni • Cucurbita pepo • Gossypium arboreum • Phaseolus vulgaris • Rhodococcus equi • Solanum lycopersicum • Zea mays |
| 17beta-estradiol | C18H24O2 | • Non-Osmolyte • Osmoregulation | • Homo sapiens |
| progesterone | C21H30O2 | • Non-Osmolyte • Osmoregulation | • Digitalis • Digitalis lanata • Digitalis purpurea • Homo sapiens |
| ethylene | C2H4 | • Osmoregulation • Non-Osmolyte | • Arabidopsis thaliana • Clostridium bifermentans • Cryptococcus albidus • Cucumis melo • Dehalobacter restrictus • Dehalococcoides mccartyi • Dehalococcoides sp. VS • Desulfitobacterium chlororespirans • Desulfitobacterium hafniense • Dianthus caryophyllus • Escherichia coli • Glycine max • Malus domestica • Penicillium digitatum • Pseudomonas syringae • Ralstonia solanacearum • Saccharomyces cerevisiae • Solanum lycopersicum • Sulfurospirillum multivorans • Synechococcus elongatus • Synechocystis sp. PCC 6803 • Vigna radiata |
| gibberellin A3 | C19H21O6 | • Osmoregulation | • Arabidopsis thaliana • Brassica juncea • Carthamus tinctorius • Fusarium fujikuroi • Glycine max • Homo sapiens • Linum usitatissimum • Oryza sativa • Pisum sativum • Sesamum indicum • Spinacia oleracea • Thunbergia alata • Triticum aestivum |
| (-)-jasmonate | C12H17O3 | • Osmoregulation • Non-Osmolyte | • Arabidopsis thaliana • Nicotiana attenuata • Oryza sativa • Solanum lycopersicum • Solanum tuberosum |
| indole-3-acetate | C10H8N1O2 | • Osmoregulation • Non-Osmolyte | • Agrobacterium • Agrobacterium tumefaciens • Alcaligenes faecalis • Arabidopsis thaliana • Azospirillum brasilense • Azospirillum lipoferum • Azotobacter vinelandii • Bacillus cereus • Bacillus sp. No. 230 • Brachybacterium conglomeratum • Bradyrhizobium japonicum • Burkholderia lata • Burkholderia pyrrocinia • Catharanthus roseus • Citrus sinensis • Enterobacter cloacae • Enterococcus faecalis • Escherichia coli K4 • Homo sapiens • Marinomonas sp. MWYL1 • Mesorhizobium loti • Micrococcus percitreus  • Mus musculus • Oryza sativa • Pantoea agglomerans • Populus tremula x Populus tremuloides • Pseudomonas chlororaphis • Pseudomonas fluorescens • Pseudomonas putida • Pseudomonas savastanoi • Rattus norvegicus • Rhizobiaceae • Rhizobium leguminosarum • Rhizobium phaseoli • Rhodococcus jostii • Sinorhizobium meliloti Rm2011 • Solanum lycopersicum • Sphingomonas wittichii • Sulfolobus tokodaii • Sus scrofa • Taphrina deformans • Taphrina pruni • Taphrina wiesneri • Vicia faba • Xanthobacter agilis • Zea mays |
| 2-cis-abscisate | C15H19O4 | • Osmoregulation • Non-Osmolyte | • Arabidopsis thaliana • Hordeum vulgare • Solanum lycopersicum • Solanum tuberosum • Vigna angularis |
| all-trans-beta-carotene | C40H56 | • Osmoprotectant | • Adonis aestivalis • Adonis annua • Agrobacterium aurantiacum • Arabidopsis thaliana • Bixa orellana • Brassica juncea • Capsicum annuum • Carica papaya • Carthamus tinctorius • Crocus sativus • Glycine max • Haematococcus pluvialis • Homo sapiens • Lactuca sativa romaine • Linum usitatissimum • Mycobacterium lacticola • Narcissus pseudonarcissus • Nostoc punctiforme • Oryza sativa • Pantoea agglomerans • Pantoea ananatis • Pisum sativum • Sesamum indicum • Solanum lycopersicum • Solanum tuberosum • Spinacia oleracea • Synechococcus sp. PCC 7002 • Thunbergia alata • Xanthophyllomyces dendrorhous |
| N-acetylglutaminylglutamine amide | C12H21N5O5 | • Osmoprotectant | • Sinorhizobium meliloti |
| taurine | C2H7N1O3S1 | • Osmoprotectant | • Achromobacter superficialis • Achromobacter xylosoxidans  • Arabella iricolor • Bilophila wadsworthia • Bos taurus • Busycotypus canaliculatus • Castellaniella denitrificans • Cellana grata • Chromohalobacter salexigens • Crassostrea gigas • Desulfonispora thiosulfatigenes • Escherichia coli • Halichondria japonica • Haliotis tuberculata • Homo sapiens • Klebsiella oxytoca • Littorina littorea • Mammalia • Marphysa sanguinea • Meretrix lusoria • Mus musculus • Oryctolagus cuniculus • Paracoccus denitrificans • Patiria pectinifera • Pecten jacobaeus • Pecten maximus • Pseudomonas aeruginosa • Rattus norvegicus • Rhodococcus opacus • Scapharca broughtonii • Suberites domuncula |
| 3-(N-morpholino)propanesulfonate | C7H14N1O4S1 | • Osmoprotectant | • Escherichia coli |
| isethionate | C2H5O4S1 | • Candidate Osmoprotectant | • a non specified bacterium (Kondo 77) • Chromohalobacter salexigens • Dickeya dadantii • Escherichia coli • Klebsiella oxytoca • Marinomonas sp. MED121 • Paracoccus denitrificans • Psychromonas ingrahamii |
| glutathione | C10H16N3O6S1 | • Osmoprotectant | • Acidiphilium acidophilum • Acidithiobacillus ferrooxidans • Acidithiobacillus thiooxidans • Allochromatium vinosum • Arabidopsis thaliana • Astragalus • Blighia sapida • Bos taurus • Brassica juncea • Brassica napus • Brassica rapa • Cavia porcellus • Chlorella sp. (Roon 68) • Crithidia fasciculata • Cucumis sativus • Cucurbita pepo medullosa  • Drosophila melanogaster • Escherichia coli • Euglena • Glycine max • Homo sapiens • Lemna minor • Lotus japonicus • Musca domestica • Mus musculus • Neptunia • Oryctolagus cuniculus • Paracoccus denitrificans • Paracoccus versutus • Phanerochaete chrysosporium • Pisum sativum • Populus tremula x Populus alba • Pseudomonas cepacia • Pseudomonas putida • Pseudomonas sp GU5 • Pteris vittata • Rattus • Rattus norvegicus • Rhodobacter sphaeroides • Saccharomyces cerevisiae • Schizosaccharomyces pombe • Solanum tuberosum • Sphingobium chlorophenolicum • Sphingobium japonicum • Sphingomonas sp. SYK6 • Spinacia oleracea • Starkeya novella • Sus scrofa • Thiobacillus thioparus • Triticum aestivum |
| monodehydroascorbate radical | C6H7O6 | • Osmoregulation | • Arabidopsis thaliana • Bos taurus • Brassica rapa • Cavia porcellus • Cucumis sativus • Cucurbita pepo medullosa  • Euglena • Homo sapiens • Mus musculus • Pisum sativum • Rattus norvegicus • Solanum tuberosum • Spinacia oleracea |
| L-ascorbate | C6H7O6 | • Osmoprotectant | • Actinidia • Amanita muscaria • Apis mellifera • Arabidopsis thaliana • Bos taurus • Brassica oleracea • Brassica rapa • Caenorhabditis elegans • Capra hircus • Capsicum annuum • Cavia porcellus • Chlamydomonas reinhardtii • Corynebacterium sp. SHS 0007 • Cucumis melo • Cucumis sativus • Cucurbita pepo medullosa  • Dianthus caryophyllus • Drosophila melanogaster • Dugesia japonica • Escherichia coli • Euglena • Euglena gracilis • Euglena gracilis Z • Gluconobacter oxydans • Glycine max • Homo sapiens • Ketogulonicigenium vulgare DSM 4025 • Klebsiella pneumoniae • Lactuca sativa romaine • Malus domestica • Mesocricetus auratus • Mus musculus • Nicotiana plumbaginifolia • Oryza sativa • Pantoea agglomerans • Paul's Scarlet Climber Rose • Pelargonium crispum • Periplaneta americana • Pisum sativum • Pseudomonas fluorescens • Rattus norvegicus • Solanum lycopersicum • Solanum tuberosum • Spinacia oleracea • Sus scrofa • Vigna radiata • Vitis vinifera • Xanthophyllomyces dendrorhous |
| a poly-beta-hydroxybutyrate |  | • Osmoprotectant | • Azospirillum brasilense • Azotobacter beijerinckii • Azotobacter vinelandii • Caulobacter crescentus • Cupriavidus necator • Desmonostoc muscorum • Rhodobacter sphaeroides • Streptomyces aureofaciens • Zoogloea ramigera |
| a 1,2-diacyl-sn-glycerol 3-phosphate |  | • Osmoprotectant | • Arabidopsis thaliana • Brassica juncea • Carthamus tinctorius • Glycine max • Homo sapiens • Linum usitatissimum • Oryza sativa • Pisum sativum • Sesamum indicum • Spinacia oleracea • Thunbergia alata |
| 3-hydroxybutanoate |  | • Osmoprotectant | • Escherichia coli • Lycopersicon hirsutum • Micrococcus luteus • Saccharomyces cerevisiae • Solanum lycopersicum |
| D-fructose |  | • Osmoprotectant |  |
